# First-line camrelizumab (a PD-1 inhibitor) plus apatinib (an VEGFR-2 inhibitor) and chemotherapy for advanced gastric cancer (SPACE): a phase 1 study

**DOI:** 10.1038/s41392-024-01773-9

**Published:** 2024-03-25

**Authors:** Xiaofeng Chen, Hao Xu, Xiaobing Chen, Tongpeng Xu, Yitong Tian, Deqiang Wang, Fen Guo, Kangxin Wang, Guangfu Jin, Xiao Li, Rong Wang, Fengyuan Li, Yongbin Ding, Jie Tang, Yueyu Fang, Jing Zhao, Liang Liu, Ling Ma, Lijuan Meng, Zhiguo Hou, Rongrong Zheng, Yang Liu, Ni Guan, Bei Zhang, Shuang Tong, Shiqing Chen, Xing Li, Yongqian Shu

**Affiliations:** 1 Department of Oncology, The First Affiliated Hospital with Nanjing Medical University, Nanjing, China; 2 Gusu School, Nanjing medical University, Suzhou, China; 3 Jiangsu Key Lab of Cancer Biomarkers, Prevention and Treatment, Collaborative Innovation Center for Cancer Personalized Medicine, Nanjing Medical University, Nanjing, China; 4 Department of General surgery, The First Affiliated Hospital with Nanjing Medical University, Nanjing, China; 5 Department of Oncology, Henan Cancer Hospital Affiliated Cancer Hospital of Zhengzhou University, Zhengzhou, China; 6 Henan Engineering Research Center of Precision Therapy of Gastrointestinal Cancer & Zhengzhou Key Laboratory for Precision Therapy of Gastrointestinal Cancer, Zhengzhou, China; 7 Department of Oncology, Digestive Disease Institute & Cancer Institute of Jiangsu University, Affiliated Hospital of Jiangsu University, Zhenjiang, China; 8 Department of Oncology, Suzhou Hospital of Nanjing Medical University, Suzhou, China; 9 Department of Oncology, Nanjing PuKou People’s Hospital, Nanjing, China; 10 Department of Epidemiology, School of Public Health, Nanjing Medical University, Nanjing, China; 11 Department of Pathology, The First Affiliated Hospital with Nanjing Medical University, Nanjing, China; 12 Department of general surgery, Jurong Branch hospital of Jiangsu Province People Hospital, Jurong, China; 13 Department of Medical Oncology, Liyang People’s Hospital, Liyang, China; 14 Department of radiology, Nanjing PuKou People’s Hospital, Nanjing, China; 15 Jiangsu Hengrui Pharmaceuticals, Shanghai, China; 16 Department of Medical Affairs, 3D Medicines Inc., Shanghai, China; 17 Shanghai OrigiMed Co., Ltd., Shanghai, China

**Keywords:** Gastrointestinal cancer, Tumour immunology

## Abstract

Patients with advanced gastric cancer typically face a grim prognosis. This phase 1a (dose escalation) and phase 1b (dose expansion) study investigated safety and efficacy of first-line camrelizumab plus apatinib and chemotherapy for advanced gastric or gastroesophageal junction adenocarcinoma. The primary endpoints included maximum tolerated dose (MTD) in phase 1a and objective response rate (ORR) across phase 1a and 1b. Phase 1a tested three dose regimens of camrelizumab, apatinib, oxaliplatin, and S-1. Dose regimen 1: camrelizumab 200 mg on day 1, apatinib 250 mg every other day, oxaliplatin 100 mg/m² on day 1, and S-1 40 mg twice a day on days 1–14. Dose regimen 2: same as dose regimen 1, but oxaliplatin 130 mg/m². Dose regimen 3: same as dose regimen 2, but apatinib 250 mg daily. Thirty-four patients were included (9 in phase 1a, 25 in phase 1b). No dose-limiting toxicities occurred so no MTD was identified. Dose 3 was set for the recommended phase 2 doses and administered in phase 1b. The confirmed ORR was 76.5% (95% CI 58.8–89.3). The median progression-free survival was 8.4 months (95% CI 5.9-not evaluable [NE]), and the median overall survival (OS) was not mature (11.6-NE). Ten patients underwent surgery after treatment and the multidisciplinary team evaluation. Among 24 patients without surgery, the median OS was 19.6 months (7.8-NE). Eighteen patients (52.9%) developed grade ≥ 3 treatment-emergent adverse events. Camrelizumab plus apatinib and chemotherapy showed favorable clinical outcomes and manageable safety for untreated advanced gastric cancer (ChiCTR2000034109).

## Introduction

Gastric cancer is the fifth most frequently diagnosed cancer and the fourth leading cause of cancer-related deaths.^1^ Many patients are diagnosed at an advanced stage.^2^ The standard treatment for untreated advanced gastric cancer is platinum- or fluoropyrimidine-based chemotherapy.^2^ More recently, the addition of nivolumab to chemotherapy has become a standard regimen since the CheckMate 649 trial demonstrated longer overall survival (OS) with nivolumab plus chemotherapy compared to chemotherapy alone, with a modest but statistically significant survival benefit.^3^ However, this combination therapy proved more advantageous in patients with a PD-L1 combined positive score (CPS) ≥ 5 than those with PD-L1 CPS < 5, and is strongly recommended for patients with PD-L1 CPS ≥ 5.^4–6^ Furthermore, the KEYNOTE-859 trial demonstrated that the combination of pembrolizumab and chemotherapy led to a statistically significantly enhancement in OS, surpassing placebo alongside chemotherapy, but with a limited survival advantage.^7,8^ Current first-line treatments are inadequate, so novel therapies are urgently needed to further improve survival for advanced gastric cancer.

The abnormal vasculature in the tumor microenvironment (TME) causes immunosuppression by various mechanisms, such as increasing the number of regulatory T (Treg) cells, recruiting tumor-associated macrophages (TAMs) and inflammatory monocytes, reprogramming TAMs to pro-tumor M2 phenotype, inhibiting dendritic cells (DCs) maturation, impairing antigen presentation and activation of tumor-specific cytotoxic T lymphocytes. Anti-angiogenesis inhibitors can reverse these effects by normalizing the vasculature and recalibrating the TME towards a state that stimulates the immune response, thereby enhancing the efficacy of anti-PD1/PD-L1 antibodies.^9,10^ As a result, the strategy of pairing anti-angiogenesis inhibitors with anti-PD1/PD-L1 antibodies is a highly prospective approach for cancer treatment, and several such combinations were approved as standard therapies for different types of cancer, such as unresectable hepatocellular carcinoma (HCC), advanced renal cell carcinoma, and advanced non-squamous non-small cell lung cancer (NSCLC).^11–16^

Apatinib, a new small-molecule tyrosine kinase inhibitor, selectively focuses on vascular endothelial growth factor receptor 2 (VEGFR2), thereby restraining the angiogenesis of tumors.^17^ Following the evidence of significantly improved survival outcomes shown in phase 2 and 3 clinical trials, approval was granted in China for apatinib usage as a third- or later-line treatment for advanced gastric adenocarcinoma.^18,19^ Apatinib plus camrelizumab (an anti-PD-1 antibody) exhibited encouraging outcomes among patients with advanced NSCLC, HCC, and advanced esophageal squamous cell cancer.^20–22^ Apatinib plus camrelizumab was approved for advanced HCC in China.^23^ Furthermore, a phase 2 clinical trial administering camrelizumab alongside CAPOX, followed by a combination of camrelizumab and apatinib for untreated advanced gastric cancer, has reflected promising anti-cancer efficacy and a tolerable safety record.^24^

Notably, neoadjuvant camrelizumab plus apatinib and chemotherapy showed promising efficacy. It reduced tumor mutational burden (TMB) and altered immune cell subsets by increasing DCs, CD8^+^ T cells, and M1 phenotype TAMs in all patients. In patients with a partial pathological response, it reduced subclone diversity and Treg cells, and expanded T cell clones.^25^ These results suggest that it effectively established an immunostimulatory TME. The first interim analysis of the phase 3 trial (DRAGON IV/CAP 05) of apatinib, camrelizumab, and chemotherapy as perioperative treatment for gastric cancer revealed a significant improvement in the primary endpoint of pathological complete response.^26^ Drawing upon these encouraging results, camrelizumab plus apatinib and chemotherapy may provide a valuable treatment option for advanced gastric adenocarcinoma. This phase 1 study aimed to ascertain the optimal dosage, tolerability, and antitumor activity of camrelizumab plus apatinib and chemotherapy as a potential treatment for untreated advanced gastric cancer.

## Results

### Patients

**Fig. 1:**
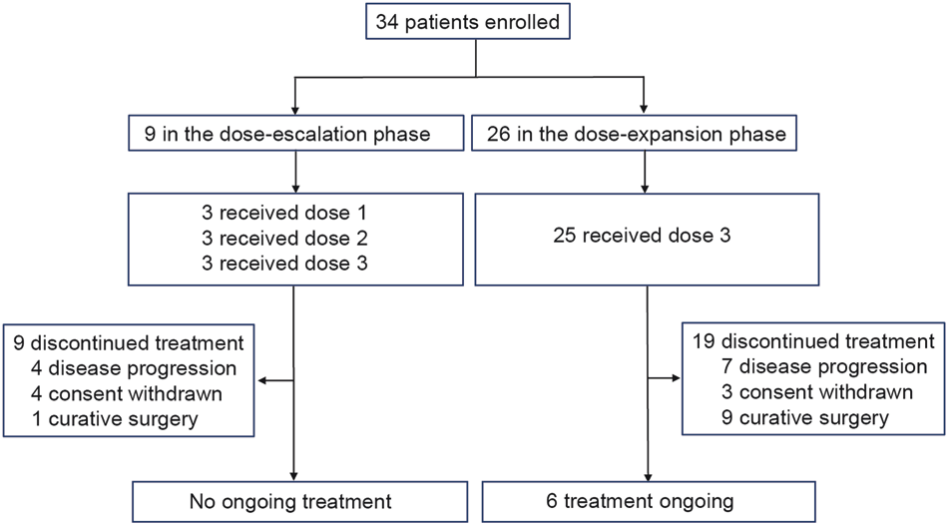
Trial profile

Between June 12, 2020, and October 6, 2022, the study enrolled 34 patients. All patients received study treatment. Nine patients were included in phase 1a, and 25 were included in phase 1b. Throughout phase 1a, nine patients terminated treatment due to voluntary withdrawal (*n* = 4), disease progression (*n* = 4), and undergoing curative surgery (*n* = 1). In phase 1b, 19 patients terminated treatment because of disease progression (*n* = 7), voluntary withdrawal (*n* = 3), and undergoing curative surgery (*n* = 9), while six patients continued treatment until the data cutoff date of February 21, 2023 (Fig. 1). The full analysis set and safety analysis set included 34 patients.

Table 1 provides a summarized overview of the patients’ baseline characteristics. The vast majority were male (94.1%), and 25 patients (73.5%) had primary tumors located in the stomach. All patients had metastases, including 33 (97.1%) with lymph node metastases, 19 (55.9%) with liver metastases, and 3 (8.8%) with peritoneal metastases. 64.7% of patients had ≥ 2 metastatic sites. 35.3% of patients had a PD-L1 CPS ≥ 1, 20.6% had CPS ≥ 5, and 14.7% had CPS ≥ 10. Moreover, 13 patients (38.2%) had high TMB. One patient had both high TMB and high microsatellite instability. The baseline characteristics of patients who were administered dose 3 closely resembled those of all patients in this study.

**Table 1:** Baseline characteristics

	**All (*n*** = **34)**	**Dose 3 (*n*** = **28)**
**Median age, years (range)**	58.5 (38.0, 75.0)	58.0 (38.0, 70.0)
**Sex,** ***n*** **(%)**		
Male	32 (94.1)	26 (92.9)
Female	2 (5.9)	2 (7.1)
**ECOG performance status,** ***n*** **(%)**		
0	15 (44.1)	13 (46.4)
1	19 (55.9)	15 (53.6)
**Primary tumor location at diagnosis,** ***n*** **(%)**		
Gastroesophageal junction	9 (26.5)	8 (28.6)
Gastric	25 (73.5)	20 (71.4)
**Histology,** ***n*** **(%)**		
Intestinal type	14 (41.2)	11 (39.3)
Diffuse type	5 (14.7)	2 (7.1)
Mixed	7 (20.6)	7 (25.0)
Unknown	8 (23.5)	8 (28.6)
**Differentiation type,** ***n*** **(%)**		
Low	19 (55.9)	16 (57.1)
Intermediate	11 (32.4)	8 (28.6)
Unknown	4 (11.8)	4 (14.3)
**Number of organs with metastases**		
≤1	12 (35.3)	10 (35.7)
≥2	22 (64.7)	18 (64.3)
**Microsatellite status,** ***n*** **(%)**		
Microsatellite stable (MSS)	30 (88.2)	24 (85.7)
Microsatellite instability (MSI)-high	1 (2.9)	1 (3.6)
MSI-low	1 (2.9)	1 (3.6)
Unknown	2 (5.9)	2 (7.1)
**Tumor mutational burden (TMB),** ***n*** **(%)**		
TMB-high	13 (38.2)	11 (39.3)
TMB-low	19 (55.9)	15 (53.6)
Unknown	2 (5.9)	2 (7.1)
**PD-L1 combined positive score (CPS),** ***n*** **(%)**		
< 1	17 (50.9)	12 (42.9)
≥ 1	12 (35.3)	11 (39.3)
< 5	22 (64.7)	17 (60.7)
≥ 5	7 (20.6)	6 (21.4)
< 10	24 (70.6)	19 (67.9)
≥ 10	5 (14.7)	4 (14.3)
Unknown	5 (14.7)	5 (17.9)

TMB-high was defined as TMB ≥ 10 mutants/megabase. TMB-low was defined as TMB < 10 mutants/megabase

### Recommended phase 2 doses (RP2Ds) and safety

In phase 1a, no dose-limiting toxicities (DLTs) were observed across all dose regimens, thus no maximum tolerated dose (MTD) was identified. Three patients each at dose 1 and 3 had a partial response; two of three patients at dose 2 also had a partial response (one lacked post-baseline response evaluation data). Dose 3 used standard chemotherapy, camrelizumab, and apatinib doses in clinical practice. It showed tolerable safety and promising antitumor activity. Thus, we chose dose 3 as the RP2Ds.

In the safety analysis set, all patients experienced treatment-emergent adverse events (TEAEs) of any grade, with 18 (52.9%) experiencing TEAEs of grade 3 or above. Nineteen patients (55.9%) experienced immune-related adverse events (irAEs), but only five (14.7%) experienced irAEs of grade 3 or above (Table 2). Furthermore, TEAEs resulted in dose delay or interruption in 19 patients (55.9%), mainly including hepatic dysfunction (4 [11.8%]) and decreased platelet count (4 [11.8%]). TEAEs resulted in dosage decrease in 21 patients (61.8%), mainly including decreased platelet count (8 [23.5%]), increased alanine aminotransferase (4 [11.8%]), increased aspartate aminotransferase (4 [11.8%]) and decreased neutrophil count (4 [11.8%]) (Supplementary Table 1). One patient died due to gastrointestinal bleeding unrelated to treatment.

**Table 2:** Adverse events

	All (*n* = 34)			Dose 3 (*n* = 28)		
Treatment-emergent adverse events (TEAEs), *n* (%)	Any grade	Grade 1–2	Grade ≥ 3	Any grade	Grade 1–2	Grade ≥ 3
Any TEAE	34 (100.0)	16 (47.1)	18 (52.9)	28 (100.0)	13 (46.4)	15 (53.6)
Decreased platelet count	16 (47.1)	14 (41.2)	2 (5.9)	14 (50.0)	13 (46.4)	1 (3.6)
Decreased neutrophil count	15 (44.1)	9 (26.5)	6 (17.6)	14 (50.0)	8 (28.6)	6 (21.4)
Increased aspartate aminotransferase	14 (41.2)	13 (38.2)	1 (2.9)	13 (46.4)	12 (42.9)	1 (3.6)
Decreased white blood cell count	13 (38.2)	12 (35.3)	1 (2.9)	11 (39.3)	10 (35.7)	1 (3.6)
Increased alanine aminotransferase	12 (35.3)	11 (32.4)	1 (2.9)	11 (39.3)	10 (35.7)	1 (3.6)
Hypertension	12 (35.3)	11 (32.4)	1 (2.9)	10 (35.7)	10 (35.7)	0
Fatigue	11 (32.4)	11 (32.4)	0	9 (32.1)	9 (32.1)	0
Neurotoxicity	11 (32.4)	11 (32.4)	0	7 (25.0)	7 (25.0)	0
Decreased appetite	11 (32.4)	11 (32.4)	0	9 (32.1)	9 (32.1)	0
Reactive cutaneous capillary endothelial proliferation	10 (29.4)	10 (29.4)	0	7 (25.0)	7 (25.0)	0
Anemia	10 (29.4)	9 (26.5)	1 (2.9)	8 (28.6)	7 (25.0)	1 (3.6)
Proteinuria	9 (26.5)	8 (23.5)	1 (2.9)	7 (25.0)	7 (25.0)	0
Hypoalbuminemia	9 (26.5)	9 (26.5)	0	8 (28.6)	8 (28.6)	0
Vomiting	9 (26.5)	8 (23.5)	1 (2.9)	8 (28.6)	7 (25.0)	1 (3.6)
Increased blood bilirubin	9 (26.5)	9 (26.5)	0	8 (28.6)	8 (28.6)	0
Hand-foot syndrome	8 (23.5)	8 (23.5)	0	6 (21.4)	6 (21.4)	0
Diarrhea	7 (20.6)	6 (17.6)	1 (2.9)	6 (21.4)	5 (17.9)	1 (3.6)
Hepatic function abnormal	6 (17.6)	1 (2.9)	5 (14.7)	6 (21.4)	1 (3.6)	5 (17.9)

Immune-related adverse events (irAEs), *n* (%)	Any grade	Grade 1–2	Grade ≥ 3	Any grade	Grade 1–2	Grade ≥ 3
Any irAE	19 (55.9)	14 (41.2)	5 (14.7)	16 (57.1)	12 (42.9)	4 (14.3)
Reactive cutaneous capillary endothelial proliferation	10 (29.4)	10 (29.4)	0	7 (25.0)	7 (25.0)	0
Rash	5 (14.7)	2 (5.9)	3 (8.8)	5 (17.9)	2 (7.1)	3 (10.7)
Hypothyroidism	4 (11.8)	4 (11.8)	0	3 (10.7)	3 (10.7)	0
Hyperthyroidism	2 (5.9)	2 (5.9)	0	1 (3.6)	1 (3.6)	0
Immune-mediated hepatitis	2 (5.9)	0	2 (5.9)	1 (3.6)	0	1 (3.6)
Immune-related pancreatitis	1 (2.9)	1 (2.9)	0	1 (3.6)	1 (3.6)	0
Cushing’s syndrome	1 (2.9)	1 (2.9)	0	1 (3.6)	1 (3.6)	0
Pituitary dysfunction	1 (2.9)	1 (2.9)	0	1 (3.6)	1 (3.6)	0
Elevated cardiac necrosis markers	1 (2.9)	1 (2.9)	0	0	0	0

### Antitumor activity

**Fig. 2:**
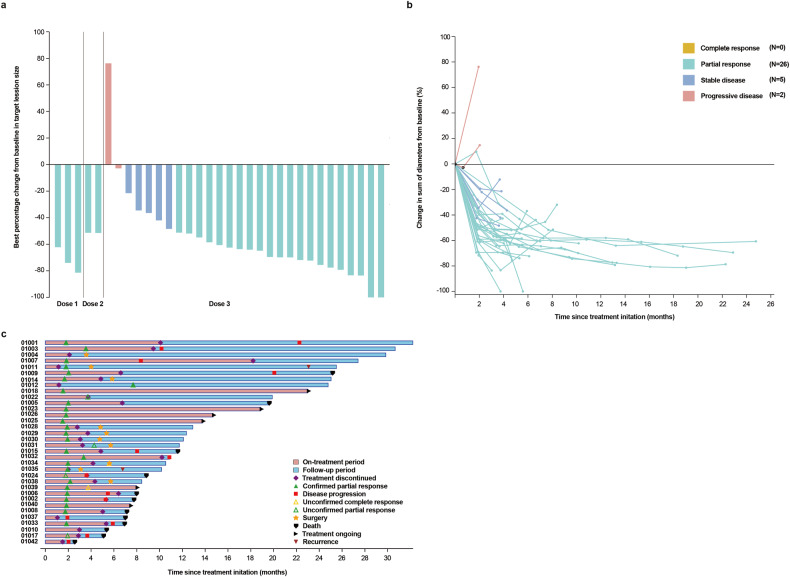
Tumor response. **a** Waterfall plot of maximum percent change in tumor size from baseline in each patient as measured by Response Evaluation Criteria in Solid Tumors (version 1.1). **b** Longitudinal percentage change in tumor size from baseline. **c** Time on treatment. Among the patients who received dose 2, one patient did not undergo post-baseline response evaluation and thus was not included in Fig. 2a, b

In the full analysis set, the confirmed objective response rate (ORR) reached 76.5% (95% confidence interval [CI] 58.8–89.3), the disease control rate (DCR) attained 91.2% (76.3–98.1), and the median duration of response (DOR) extended 7.6 months (5.4-not evaluable [NE]). The confirmed ORR stood at 70.6% (95% CI 44.0–89.7) for PD-L1 CPS < 1 and 91.7% (61.5–99.8) for PD-L1 CPS ≥ 1. Additionally, the confirmed ORR reached 100% (95% CI 59.0–100.0) for PD-L1 CPS ≥ 5 and 72.7% (49.8–89.3) for PD-L1 CPS < 5 (Table 3). For dose 3 (including phases 1a and 1b), 21 patients achieved a partial response, five had stable disease, and two had progressive disease (PD). More detailed information regarding individual response and treatment status during the study is provided in Fig. 2. Subgroup analysis showed higher confirmed ORR in patients with gastric cancer, TMB-high, Eastern Cooperative Oncology Group (ECOG) performance status (ps) of 0, or PD-L1 CPS ≥ 1, in contrast to individuals with gastroesophageal junction (GEJ) cancer, TMB-low, ECOG ps 1, or PD-L1 CPS < 1. Among three patients with peritoneal metastases, none responded to combined therapy (Supplementary Fig. 1).

**Table 3:** Tumor response assessed by Response Evaluation Criteria in Solid Tumors (version 1.1)

	Full analysis set (*n* = 34)	PD -L1 CPS < 1 (*n* = 17)	PD -L1 CPS ≥ 1 (*n* = 12)	PD -L1 CPS < 5 (*n* = 22)	PD -L1 CPS ≥ 5 (*n* = 7)
Partial response, *n* (%)	26 (76.5)	12 (70.6)	11 (91.7)	16 (72.7)	7 (100.0)
Stable disease, *n* (%)	5 (14.7)	2 (11.8)	1 (8.3)	3 (13.6)	0
Progressive disease, *n* (%)	2 (5.9)	2 (11.8)	0	2 (9.1)	0
Not evaluable, *n* (%)	1 (2.9)	1 (5.9)	0	1 (4.5)	0
Confirmed objective response rate, % (95% CI)	76.5 (58.8–89.3)	70.6 (44.0–89.7)	91.7 (61.5–99.8)	72.7 (49.8–89.3)	100.0 (59.0–100.0)

*CPS* combined positive score

**Fig. 3:**
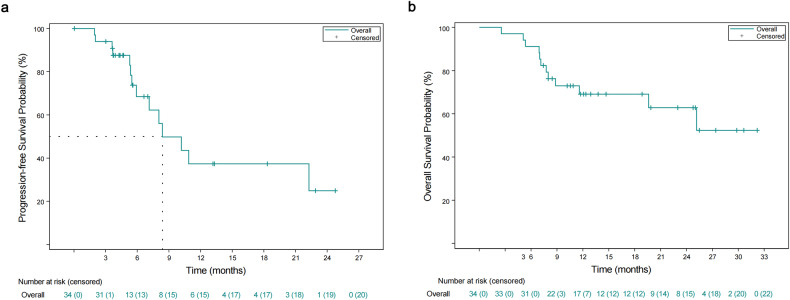
Survival outcomes in all treated patients. **a** Progression-free survival. **b** Overall survival. Among ten surgical patients, censoring was performed on the last imaging before surgery

In the full analysis set, over a median follow-up period of 11.9 months (IQR, 8.0-23.0), 14 patients experienced PD or death, and the median progression-free survival (PFS) stood at 8.4 months (95% CI 5.9-NE). Twelve patients died, and the median OS was not mature (95% CI 11.6-NE). The rates of OS at one year and two years reached 69.1% (95% CI 49.9–82.2) and 62.8% (41.3–78.3), respectively (Fig. 3). Sixteen patients experienced PD, recurrence or death, leading to a median event-free survival (EFS) of 22.3 months (95% CI 7.1-NE). Additionally, the median EFS was 23.1 months (95% CI 6.8-NE) with surgery, contrasted with 8.4 months (5.5–22.3) without surgery. The median OS was not mature with surgery, contrasted with 19.6 months (95% CI 7.8-NE) without surgery (Supplementary Fig. 2). Furthermore, detailed pathological outcomes for the ten patients who underwent surgery are presented in Supplementary Table 2.

### Exploratory analyses

A total of 29 baseline samples and seven surgical samples (including four paired baseline-surgical samples) were assessed for the tumor immune microenvironment using multiplex immunofluorescence staining. After treatment, the proportion of patients with tertiary lymphoid structure (TLS) increased significantly compared to baseline samples (Supplementary Fig. 3a, *p* = 0.03). Among the four paired baseline-surgical samples, three samples lacking TLS at baseline developed TLS after treatment; one pair remained TLS-negative. Moreover, reductions in immune infiltration were observed after treatment, including tumoral PD1^+^ (*p* = 0.025), CD3^+^ (*p* = 0.030), Foxp3^+^ (*p* = 0.034), M2 Macrophages (*p* = 0.031), M1 macrophages (*p* = 0.010) cell density, as well as stromal PD1^+^ (*p* = 0.003), M1 macrophage (*p* < 0.001), CD20^+^ (*p* = 0.018), Foxp3^+^ cell (*p* = 0.003) cell density. However, no significant differences were found among the paired samples. Finally, using median as cutoff, patients with high baseline tumoral CD3^+^ and Foxp3^+^ cell density had longer OS (not mature vs 19.6 months, hazard ratio [HR] 0.20, 95% CI 0.04–0.91, *p* = 0.021; not mature vs 19.6 months, HR 0.25, 95% CI 0.05-1.15, *p* = 0.054) (Supplementary Fig. 3b-c).

## Discussion

This is the first study of PD-1 blockade combined with anti-angiogenic drug and chemotherapy for untreated advanced gastric cancer. Since no MTD was observed, dose 3 was determined to be RP2Ds. Among the 34 patients who were administered the study treatment, the confirmed ORR stood at 76.5%, the median PFS reached 8.4 months, the median EFS reached 22.3 months, and the 2-year OS rate stood at 62.8%. As a result of the multidisciplinary team (MDT) evaluation, ten patients proceeded to surgical intervention. Surgically treated patients had longer survival outcomes versus non-surgical patients (median EFS: 23.1 vs 8.4 months; median OS not reached vs 19.6 months). The combination therapy exhibited a manageable safety profile.

In the present study, we observed that 35.3% of patients presented with PD-L1 CPS ≥ 1, 20.6% indicated CPS ≥ 5, and 14.7% recorded CPS ≥ 10. These percentages fall below those reported in the CheckMate 649 trial (82.0% for CPS ≥ 1 and 60.4% for CPS ≥ 5) and the KEYNOTE-859 trial (78.2% for CPS ≥ 1 and 34.9% for CPS ≥ 10).^3,8^ However, the confirmed ORR in this study surpassed numerically the results of the nivolumab or pembrolizumab plus chemotherapy groups in the CheckMate 649 trial (58%) and the KEYNOTE-859 trial (51.3%).^3,7^ A phase 2 study evaluating camrelizumab alongside chemotherapy followed by camrelizumab plus apatinib for untreated advanced gastric cancer reported a confirmed ORR of 58.3%.^24^ Additionally, nivolumab plus chemotherapy yielded a median OS of 13.8 months in the CheckMate 649 trial and 17.5 months in the ATTRACTION-4 trial. In the KEYNOTE-859 and ORIENT-16 trials, pembrolizumab or sintilimab combined with chemotherapy yielded a median OS of 12.9 and 15.2 months, respectively.^3,7,27,28^ In contrast, this study showed substantial clinical benefits. Therefore, the integration of apatinib with camrelizumab and chemotherapy could potentially enhance both short-term and long-term results.

Notably, the addition of apatinib to camrelizumab plus chemotherapy demonstrated encouraging efficacy, specifically among the group with low PD-L1 CPS scores. The combination therapy yielded a confirmed ORR in the PD-L1 CPS < 1 (70.6%) and CPS < 5 (72.7%) subsets. In contrast, the confirmed ORR was 51% and 55% for these subgroups in the CheckMate 649 trial.^3^ Moreover, peritoneal metastasis is an adverse prognostic factor. Unfortunately, none of the patients with peritoneal metastasis showed tumor reduction with combination therapy.

Given the poor prognosis of patients with late-stage gastric cancer, multiple studies investigated curative surgery as a potential solution for cases with limited non-curable factors.^29–31^ The phase 3 REGATTA study recruited individuals diagnosed with clinical stage IV and a single incurable metastasis (located in either peritoneum, liver, or para-aortic lymph nodes). The study did not demonstrate a survival advantage from palliative gastrectomy followed by chemotherapy compared to chemotherapy alone.^30^ In the AIO-FLOT3 study, 60 patients with limited metastatic gastric cancer received induction chemotherapy followed by surgery with the aim of curing or prolonging their lives. Among 36 patients who were given curative surgery, the R0 resection rate stood at 80.6%, and these patients exhibited enhanced survival relative to patients who did not receive surgery.^31^ However, the impact of curative surgery in managing metastatic gastric cancer remains unclear, particularly regarding suitable candidates and optimal treatment approaches before surgery. In this study, ten patients received combination therapy with camrelizumab, apatinib, and chemotherapy followed by curative surgery. Of these patients, nine had distant lymph node metastases and five had liver metastases. Impressively, 80% of patients achieved a partial response to the combination regimen, and 90% attained R0 resection. Pathological complete responses (pCR) were observed in three patients (30%), while five patients (50%) achieved major pathological responses (MPR). These findings are comparable to a phase 2 clinical trial of neoadjuvant camrelizumab plus apatinib and chemotherapy for treatment of resectable gastric cancer, which reported an ORR of 28.6%, an R0 resection of 72.7%, and a pCR and MPR of 12.5% each, among 14 patients with cT4bN+ clinical stage, which overlapped with inclusion criteria in this study.^25^

In a subset of patients, multiplex immunofluorescence analysis demonstrated a significant increase in TLS after treatment. TLS was linked to improved outcomes and heightened response to immune checkpoint inhibitors in cancer treatment.^32–34^ This finding may underpin the favorable efficacy of the combination therapy. Elevated infiltration of CD3^+^ or Foxp3^+^ T cells was linked to improved survival in gastric cancer.^35–37^ Similarly, in this study, patients receiving combination therapy exhibited an association of elevated levels of CD3^+^ cells or Foxp3^+^ cells with extended OS. However, the study failed to definitively identify specific immune cell types. In colon cancer, Foxp3^+^ Tregs were classified into two subsets: CD38^+^Foxp3^+^ Tregs linked to negative prognosis, and CD38^-^Foxp3^+^ Tregs linked to positive survival.^38^ Given the distinct functions of each immune cell type, further research is crucial for accurately interpreting the immune results highlighted in this study.

The safety characteristics identified in this study were in line with those detailed in the phase 2 clincal trial investigation of neoadjuvant camrelizumab in combination with apatinib and chemotherapy for locally advanced gastric cancer.^25^ Additionally, it was consistent with the phase 2 study of first-line camrelizumab plus chemotherapy followed by camrelizumab and apatinib for advanced gastric cancer.^24^ The latter phase 2 study reported that 68.8% of patients developed treatment-related adverse events (TRAEs) of grade 3 or above, 75.0% experienced treatment interruption because of TRAEs, and 41.7% experienced dosage decrease because of TRAEs. In comparison, in this study, TEAEs grade 3 or above were experienced by 52.9% of patients, TEAEs resulted in dose delay or interruption in 55.9% of patients, and TEAEs resulted in dosage decrease in 61.8% of patients. Thus, preliminary results suggest that this combination regimen may be a tolerable treatment approach.

This phase 1 trial investigated the dosage, safety, and antitumor activity of camrelizumab plus apatinib and chemotherapy for a small number of participants. Additionally, without pharmacokinetic and pharmacodynamic analyses, this study did not succeed in shedding light on the pharmacological processes of the combined treatment. Furthermore, biomarker analysis was performed on a subset of patients, and only relied on multiplex immunofluorescence staining without comprehensive molecular analyses such as transcriptomic or proteomic profiling. As such, the study’s findings warrant careful and prudent interpretation. Further studies with larger sample sizes, control groups, and more extensive molecular analyses are necessary to fully evaluate the efficacy and potential biomarkers of this combined treatment.

In conclusion, camrelizumab plus apatinib and chemotherapy demonstrated a tolerable safety profile in patients with untreated advanced gastric cancer. Additionally, this combination therapy exhibited antitumor activity, including a notable rate ORR and extended PFS and OS. Notably, patients exhibiting lower PD-L1 expression (CPS < 1 and CPS < 5) also achieved a high ORR. Nevertheless, additional clinical trials are indispensable for confirming the efficacy of this combination therapy.

## Methods

### Patients

This single-arm, multicenter, open-label, phase 1 trial investigated the safety and antitumor activity of camrelizumab plus apatinib and chemotherapy for untreated advanced gastric or GEJ adenocarcinoma. The study consisted of dose-escalation (phase 1a) and dose-expansion (phase 1b). The institutional review board at Jiangsu Province Hospital approved the study protocol and informed consent form. The study was carried out following the protocol, adhering to the principles of Good Clinical Practice guidelines. Prior to enrollment, all patients furnished written consent.

The study enrolled patients between the ages of 18 and 75 who had histologically or cytologically confirmed treatment-naïve gastric or GEJ adenocarcinoma, with negative or unknown HER2 status and clinical stage IV disease. Clinical stage IV included advanced metastatic gastric cancer (cTanyNanyM1) and unresectable locally advanced gastric cancer (cT4bNanyM0). Patients needed to possess at least one measurable lesion according to the RECIST version 1.1, an ECOG ps of 0–1, and an anticipated survival duration of at least three months. Adequate organ function was mandatory for enrollment, including: hemoglobin ≥ 90 g/L; neutrophil count > 1.5 × 10^9^/L; platelet count ≥ 100 × 10^9^/L; total bilirubin ≤ 1.5 × the upper limit of normal (ULN); alanine aminotransferase and aspartate aminotransferase ≤ 2.5 × ULN, or ≤ 5 × ULN if liver metastases were present; endogenous creatinine clearance ≥ 60 mL/min (Cockcroft-Gault formula); ventricular ejection fraction ≥ 50% on echocardiography assessment; and thyroid function (thyroid stimulating hormone and free thyroxine) within normal range or mildly abnormal without clinical significance. Patients who relapsed > 6 months after adjuvant chemotherapy and had no current adverse events of grade 3 or above per Common Terminology Criteria for Adverse Events (CTCAE) version 5.0 were allowed to be enrolled.

Patients with a prior occurrence of bleeding or a grade 3 or higher bleeding event within four weeks prior to screening, central nervous system metastases, uncontrolled hypertension, previous use of immunotherapies or targeted therapies, a history of immunodeficiency, organ transplant, or autoimmune diseases, or conditions such as pneumonia, pneumonitis, interstitial lung disease, or other conditions requiring steroid treatment were excluded from the study.

### Procedures

In phase 1a, the MTD was determined using a 3 + 3 dose escalation approach. Three patients were sequentially enrolled at each of the three dose levels (1, 2, and 3). At dose 1, the regimen comprised apatinib administered orally at 250 mg every other day (qod), camrelizumab administered intravenously at 200 mg on day 1, oxaliplatin administered intravenously at 100 mg/m² on day 1, and S-1 administered orally at 40 mg twice daily (bid) on days 1–14. Dose 2 involved apatinib administered orally at 250 mg qod, camrelizumab administered intravenously at 200 mg on day 1, oxaliplatin administered intravenously at 130 mg/m² on day 1, and S-1 administered orally at 40 mg bid on days 1–14. Dose 3 entailed apatinib administered orally once daily (qd) at 250 mg, camrelizumab administered intravenously at 200 mg on day 1, oxaliplatin administered intravenously at 130 mg/m² on day 1, and S-1 administered orally at 40 mg bid on days 1–14. If the number of patients who experienced DLTs within the first 21 days of treatment at each dose level was equal to or < 1, the dose level was escalated to the subsequent higher level. However, if DLTs were experienced by two or more patients at any given dosage level, the escalation process was halted and the preceding dosage level was deemed to be the MTD. A DLT was characterized as any grade 4 hematologic adverse events or any grade 3 or higher non-hematologic adverse events during the first 21 days of treatment or any adverse events of camrelizumab or apatinib that caused a delay in dosage of 21 days or more. The RP2Ds of study treatments were established by considering all accessible safety and efficacy data from phase 1a. The RP2Ds were given in phase 1b. Patients received the RP2Ds every three weeks as a treatment cycle in phase 1b. Patients were administered treatment until disease progression, intolerable toxicity, withdrawal of consent, or upon reaching eight cycles of chemotherapy and the 2-year duration for camrelizumab and apatinib.

Tumor response evaluation via computed tomography or magnetic resonance imaging occurred every six weeks throughout the initial four months of treatment, followed by assessments every 12 weeks thereafter, using RECIST 1.1 criteria. Patients who achieved a complete response or partial response were confirmed through reexamination four weeks after the first assessment. Patients with progressive disease may continue treatment beyond initial progression if the investigator determined there was continued clinical benefit. These patients were re-assessed after an additional four weeks of therapy.

For patients who were willing to undergo therapy, the MDT evaluation was conducted. Throughout the treatment, adverse events were rigorously recorded and this continued for 90 days post the final treatment session. CTCAE version 5.0 was used as a standard to evaluate the severity of these adverse events. Following discontinuation of treatment, survival monitoring took place quarterly, until the patient’s death or an instance of consent withdrawal.

### Outcomes

The primary endpoints included MTD in phase 1a and confirmed ORR for all patients treated in both phase 1a and phase 1b. The confirmed ORR was defined as the proportion of patients achieving confirmed complete response and partial response assessed by RECIST 1.1 criteria. Secondary endpoints included PFS, OS, DCR, and DOR across phase 1a and phase 1b. Exploratory endpoints consisted of analyses of biomarkers associated with antitumor activity. EFS was analyzed by post-hoc. PFS was the interval from the initial study treatment to disease progression or death from any reason. OS referred to the duration from the first study treatment to the occurrence of patient death, attributable to any cause. DCR was the proportion of patients reaching confirmed complete response, partial response, and stable disease assessed by RECIST 1.1 criteria. DOR represented the duration from the initial confirmation of complete or partial response to the initial evaluation indicating disease progression or death for any reason. EFS denoted the duration from the commencement of the first study treatment to the first instance of disease progression, disease recurrence, or death for any cause.

Antitumor activity was conducted in the full analysis set, including patients who were enrolled and administered the study treatment. Safety was conducted in the safety analysis set, including enrolled patients who received the study treatment and possessed safety records.

### Statistical analysis

In the KEYNOTE-062 study, chemotherapy (cisplatin plus 5-FU) in combination with pembrolizumab had an ORR of 48.6% in patients with PD-L1 CPS > 1 and 52.5% in patients with CPS > 10. Supposing an ORR of 50% for camrelizumab plus apatinib and chemotherapy, it is anticipated that the ORR would be elevated to 70%. With one-sided α set at 0.05 and β at 0.2, calculations rendered a sample size of 37 patients. When factoring an anticipated dropout rate of 10%, it was determined that the final sample size necessary would be 42 patients.

The ORR and DCR, along with their 95% CIs, were determined using the Clopper-Pearson method. PFS, EFS, and OS curves were generated via the Kaplan-Meier method, and their 95% CIs were computed using the Brookmeyer-Crowley method. Subgroup analysis of ORR was performed based on baseline patient characteristics. Exploratory analyses were performed to evaluate the association between immune cell infiltration levels and efficacy. The levels of immune cell infiltration were measured and classified into two categories of high and low, using the median value as the cut-off point. The survival curves of the different subgroups of patients were examined and contrasted employing the log-rank test. The Fisher’s exact test was utilized to analyze and compare categorical variables. The unpaired Wilcoxon test was utilized for comparisons of unlinked continuous variables, while the Wilcoxon paired test was applied for related samples. All statistical examinations were performed with the use of SAS 9.2 and R 4.3.0, implementing a two-sided test, and a *p*-value < 0.05 was deemed to indicate statistical significance.

### Supplementary information

supplemetary

protocol

## Data Availability

The corresponding author can provide the data upon request made in a reasonable manner.
