# Supplementary material for: First-line camrelizumab (a PD-1 inhibitor) plus apatinib (an VEGFR-2 inhibitor) and chemotherapy for advanced gastric cancer (SPACE): a phase 1 study
Source: Signal Transduct Target Ther. 2024 Mar 25;9:73. doi: 10.1038/s41392-024-01773-9 (PMC10963362; doi:10.1038/s41392-024-01773-9)
Supplement: Supplementary file 1 — supplemetary [file 41392_2024_1773_MOESM1_ESM.docx]

**Supplementary Materials for**

**First-line camrelizumab (a PD-1 inhibitor) plus apatinib (an VEGFR-2 inhibitor) and chemotherapy for advanced gastric cancer (SPACE): a phase 1 study**

Xiaofeng Chen^1,2,3*^, Hao Xu^4*^, Xiaobing Chen^5,6*^, Tongpeng Xu^1*^, Yitong Tian^1^, Deqiang Wang^7^, Fen Guo^8^, Kangxin Wang^9^, Guangfu Jin^10^, Xiao Li^11^, Rong Wang^1^, Fengyuan Li^4^, Yongbin Ding^12^, Jie Tang^13^, Yueyu Fang^9^, Jing Zhao^5^, Liang Liu^14^, Ling Ma^1^, Lijuan Meng^1^, Zhiguo Hou^15^, Rongrong Zheng^15^, Yang Liu^15^, Ni Guan^15^, Bei Zhang^16^, Shuang Tong^16^, Shiqing Chen^16^, Xing Li^17^, Yongqian Shu^1,2#^

**Correspondence to:**

Yongqian Shu, Department of Oncology, The First Affiliated Hospital with Nanjing Medical University, Nanjing, China.

Email: shuyongqian@csco.org.cn

**This PDF file includes:**

Materials and methods

Figures. S1 to S3

Tables S1 to S3

**Materials and methods**

**PD-L1 expression**

PD-L1 expression was defined as the number of PD-L1 positive cells (including tumor cells, lymphocytes and macrophages) in tumor tissues divided by the total number of tumor cells, then multiplied by 100. It was assessed by immunohistochemistry using the 22C3 pharmDx assay (Agilent Technologies, Carpinteria, CA, USA).

**Next-generation sequencing detection**

Formalin-fixed, paraffin-embedded tissue specimens were analyzed by next-generation sequencing, which targeted all the exons of 706 genes and introns of 39 genes with an average coverage of at least 1000×. Determination of microsatellite instability (MSI) and tumor mutational burden (TMB) was performed using bioinformatics approaches.^1^ MSI-high status was characterized by the presence of more than 15% of selected microsatellite loci exhibiting instability compared to corresponding peripheral blood samples. The TMB score for each tumor was quantified by tallying the number of somatic single nucleotide variants and small insertions/deletions per megabase (Mb) in the targeted coding region. Noncoding mutations, recurrent hotspot mutations, and known germline polymorphisms in the NCBI dbSNP database were excluded from the TMB calculation. A threshold value of 10 was employed to delineate between TMB-high and TMB-low samples.

**Multiplex immunofluorescence staining**

Primary antibodies against CD163, CD68, PD-1, PD-L1, CD3, CD4, CD8, CD56, CD20, Foxp3 and pan-cytokeratin (pan-CK) sequentially applied to FFPE tissue slides (Supplementary Table S3). After incubation with secondary antibodies, corresponding Opal fluorophores, and DAPI, the multiply stained slides were scanned using a Vectra Polaris Quantitative Pathology Imaging System (Akoya Biosciences). Images were analyzed using the APTIME software by 3D Medicines. Tumor parenchyma and stroma were differentiated by CK staining. The density of various cell subsets was quantified as the number of positively stained cells per mm^2^. Immune cell subsets in tumor and stromal regions were identified by detecting signals for markers including CD3^+^, CD3^+^CD4^+^, CD8^+^, Foxp3^+^, PD-1^+^CD8^+^, CD68^+^CD163^-^ (M1 macrophage), CD68^+^CD163^+^ (M2 macrophage), PD-L1^+^CD68^+^, CD56bright NK cell, CD56dim NK cell etc. Co-occurrence of CD3^+^ T cells and CD20^+^ B cells indicates tertiary lymphoid structure (TLS) formation.

**Reference**

1 De Roock, W. *et al.* KRAS, BRAF, PIK3CA, and PTEN mutations: implications for targeted therapies in metastatic colorectal cancer. *Lancet Oncol*. **12**, 594-603, (2011).

**Figure. S1. Subgroup analyses of objective response rate (ORR) based on baseline characteristics, assessed by Response Evaluation Criteria in Solid Tumors (version 1.1).**

**
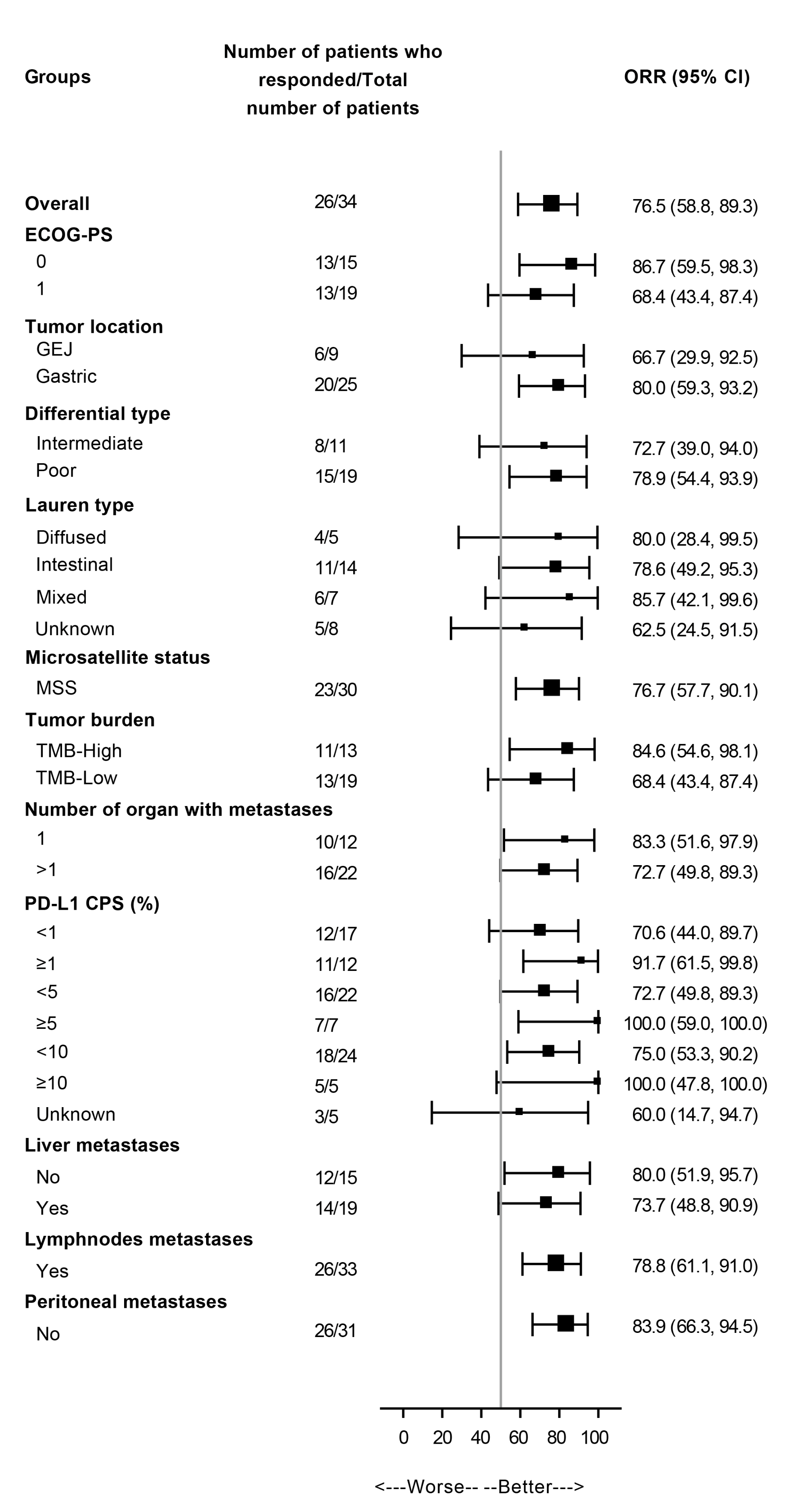
**

Three patients with peritoneal metastases did not respond, and one patient with no lymph node metastasis exhibited stable disease to the combination of camrelizumab, apatinib, and chemotherapy.

**Figure. S2. Survival outcomes.** (a) Event-free survival and (b) overall survival in all treated patients, patients who underwent surgery, and patients who did not undergo surgery.


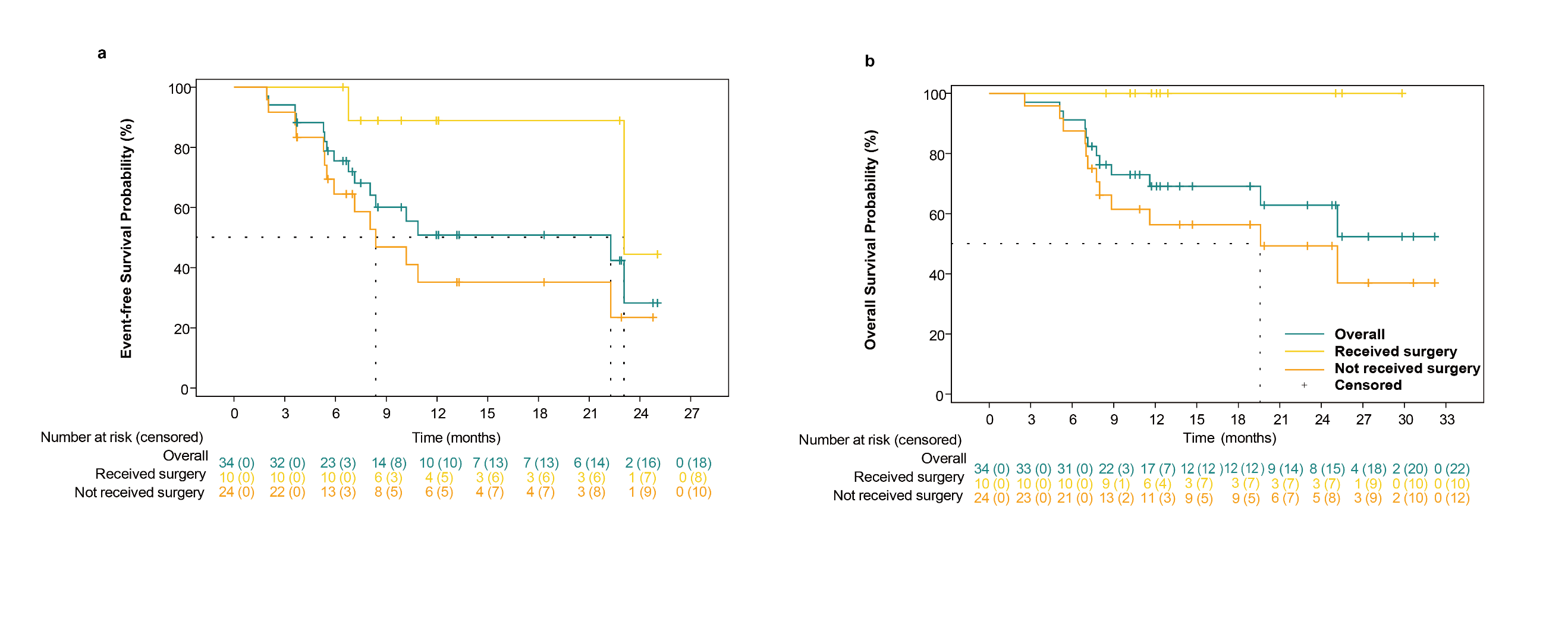


**Figure. S3. Tumor immune microenvironment analyses.** (a) Comparison of TLS between pre- and post-treatment tumors. The proportion of patients with TLS increased after treatment (p = 0.030 by Fisher's exact test). Overall survival curves based on the baseline level of CD3^+^ cells (b) and Foxp3^+^ cells (c), with the median density used as the cutoff for both.


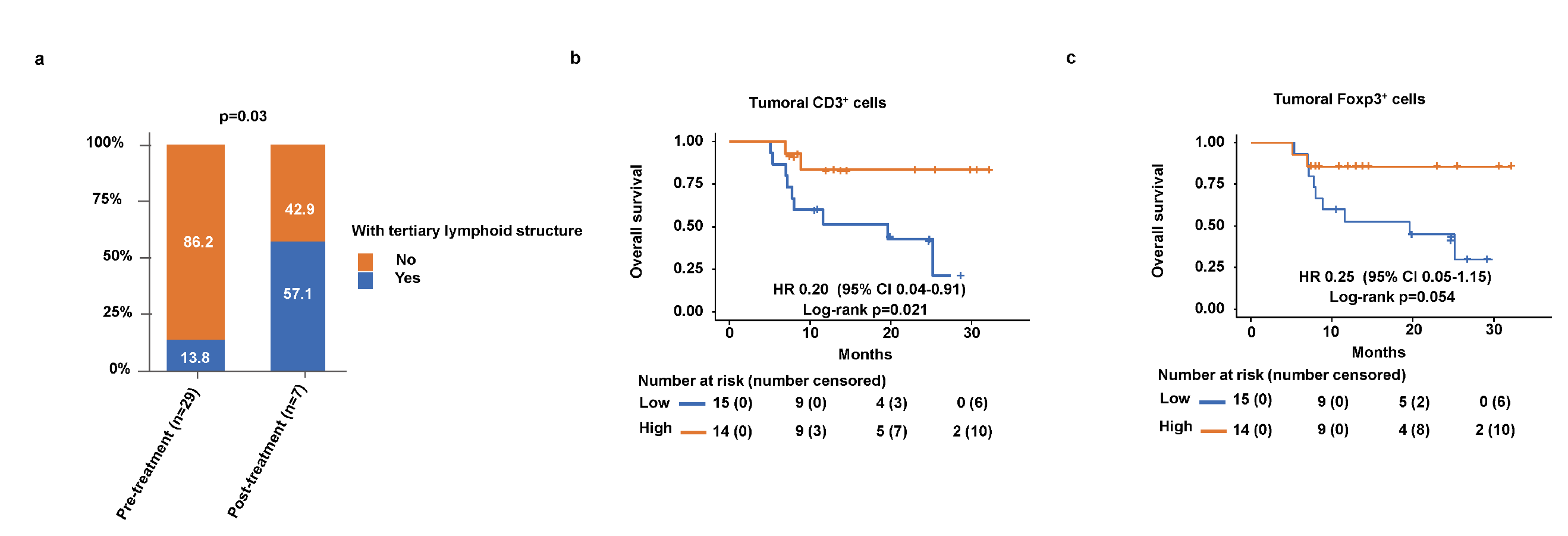


**Table S1. Treatment-emergent adverse events leading to dose interruption/delay or dose reduction.**

|  | **All (n = 34)** | | | **Dose 3 (n = 28)** | | |
| --- | --- | --- | --- | --- | --- | --- |
| **Treatment-emergent adverse events (TEAEs) leading to dose interruption/delay, n (%)** | **Any grade** | **Grade 1-2** | **Grade ≥3** | **Any grade** | **Grade 1-2** | **Grade ≥3** |
| Any TEAE leading to dose interruption/delay | 19 (55.9) | 8 (23.5) | 11 (32.4) | 15 (53.6) | 6 (21.4) | 9 (32.1) |
| Hepatic function abnormal | 4 (11.8) | 1 (2.9) | 3 (8.8) | 4 (14.3) | 1 (3.6) | 3 (10.7) |
| Decreased platelet count | 4 (11.8) | 2 (5.9) | 2 (5.9) | 3 (10.7) | 2 (7.1) | 1 (3.6) |
| Vomiting | 3 (8.8) | 3 (8.8) | 0 | 2 (7.1) | 2 (7.1) | 0 |
| Decreased neutrophil count | 3 (8.8) | 2 (5.9) | 1 (2.9) | 2 (7.1) | 1 (3.6) | 1 (3.6) |
| Proteinuria | 2 (5.9) | 1 (2.9) | 1 (2.9) | 0 | 0 | 0 |
| Immune-mediated hepatitis | 2 (5.9) | 0 | 2 (5.9) | 1 (3.6) | 0 | 1 (3.6) |
| Rash | 2 (5.9) | 0 | 2 (5.9) | 2 (7.1) | 0 | 2 (7.1) |
| Gastrointestinal haemorrhage | 2 (5.9) | 0 | 2 (5.9) | 2 (7.1) | 0 | 2 (7.1) |
| Increased alanine aminotransferase | 1 (2.9) | 1 (2.9) | 0 | 1 (3.6) | 1 (3.6) | 0 |
| Cerebral haemorrhage | 1 (2.9) | 1 (2.9) | 0 | 1 (3.6) | 1 (3.6) | 0 |
| Herpes zoster | 1 (2.9) | 1 (2.9) | 0 | 0 | 0 | 0 |
| Cholecystitis | 1 (2.9) | 1 (2.9) | 0 | 1 (3.6) | 1 (3.6) | 0 |
| Hypokalemia | 1 (2.9) | 0 | 1 (2.9) | 1 (3.6) | 0 | 1 (3.6) |
| Hyponatremia | 1 (2.9) | 1 (2.9) | 0 | 1 (3.6) | 1 (3.6) | 0 |
| Reactive cutaneous capillary endothelial proliferation | 1 (2.9) | 1 (2.9) | 0 | 0 | 0 | 0 |
| Diarrhea | 1 (2.9) | 1 (2.9) | 0 | 0 | 0 | 0 |
| Stomatitis | 1 (2.9) | 0 | 1 (2.9) | 1 (3.6) | 0 | 1 (3.6) |
| Immune-related pancreatitis | 1 (2.9) | 1 (2.9) | 0 | 1 (3.6) | 1 (3.6) | 0 |
| **TEAEs leading to dose reduction, n (%)** | **Any grade** | **Grade 1-2** | **Grade ≥3** | **Any grade** | **Grade 1-2** | **Grade ≥3** |
| Any TEAEs leading to dose reduction | 21 (61.8) | 14 (41.2) | 7 (20.6) | 20 (71.4) | 14 (50.0) | 6 (21.4) |
| Decreased platelet count | 8 (23.5) | 6 (17.6) | 2 (5.9) | 7 (25.0) | 6 (21.4) | 1 (3.6) |
| Increased alanine aminotransferase | 4 (11.8) | 4 (11.8) | 0 | 4 (14.3) | 4 (14.3) | 0 |
| Increased aspartate aminotransferase | 4 (11.8) | 4 (11.8) | 0 | 4 (14.3) | 4 (14.3) | 0 |
| Decreased neutrophil count | 4 (11.8) | 0 | 4 (11.8) | 4 (14.3) | 0 | 4 (14.3) |
| Diarrhea | 3 (8.8) | 3 (8.8) | 0 | 3 (10.7) | 3 (10.7) | 0 |
| Decreased white blood cell count | 2 (5.9) | 1 (2.9) | 1 (2.9) | 2 (7.1) | 1 (3.6) | 1 (3.6) |
| Hypoalbuminemia | 2 (5.9) | 2 (5.9) | 0 | 2 (7.1) | 2 (7.1) | 0 |
| Hepatic function abnormal | 2 (5.9) | 0 | 2 (5.9) | 2 (7.1) | 0 | 2 (7.1) |
| Vomiting | 2 (5.9) | 2 (5.9) | 0 | 2 (7.1) | 2 (7.1) | 0 |
| Decreased appetite | 2 (5.9) | 2 (5.9) | 0 | 2 (7.1) | 2 (7.1) | 0 |
| Proteinuria | 1 (2.9) | 1 (2.9) | 0 | 1 (3.6) | 1 (3.6) | 0 |
| Mouth ulceration | 1 (2.9) | 1 (2.9) | 0 | 1 (3.6) | 1 (3.6) | 0 |
| Rash | 1 (2.9) | 0 | 1 (2.9) | 1 (3.6) | 0 | 1 (3.6) |
| Neurotoxicity | 1 (2.9) | 1 (2.9) | 0 | 1 (3.6) | 1 (3.6) | 0 |
| Lower limb skin breakdown | 1 (2.9) | 1 (2.9) | 0 | 1 (3.6) | 1 (3.6) | 0 |
| Increased blood bilirubin | 1 (2.9) | 1 (2.9) | 0 | 1 (3.6) | 1 (3.6) | 0 |

**Table S2. Baseline characteristics, tumor response, and pathological response of patients who underwent surgery.**

| **Patient** | **Dose** | **cTNM** | **Distant metastatic sites** | **MSS/MSI** | **TMB** | **SOD for baseline target lesions (mm）** | **PD-L1** | **Preoperative best overall response** | **Surgery type** | **Tumor pathological regression** | **Resection** | **ypTNM Stage** | **Postoperative treatment** |
| --- | --- | --- | --- | --- | --- | --- | --- | --- | --- | --- | --- | --- | --- |
| 1 | Dose 2 | T4aN3M1 | Distant lymph nodes | MSS | TMB-Low | 16 | Unknown | Not evaluable | Unknown | Grade 2 | R0 | Stage II^1^ | None |
| 2 | Dose 3 | T3N3M1 | Distant lymph nodes | MSS | TMB-Low | 31.8 | CPS < 1 | Partial response | D2 | Grade 3 | R0 | Stage III^2^ | Two cycles of sequential SOX regimen with S-1. |
| 3 | Dose 3 | T4bN2M1 | Liver | MSS | TMB-High | 54.9 | CPS < 1 | Partial response | D2 | Grade 0 | R0 | 0^3^ | Two cycles of sequential SOX regimen followed by camrelizumab plus apatinib |
| 4 | Dose 3 | T4aN3M1 | Distant lymph nodes | MSS | TMB-Low | 21.7 | Unknown | Partial response | D2 | Grade 3 | R1 | Stage IV | Two cycles of SOX regimen plus camrelizumab and apatinib |
| 5 | Dose 3 | T3N2M1 | Liver | Unknown | Unknown | 98.1 | Unknown | Partial response | D2 | Grade 0 | R0 | Stage IV | Three cycles of sequential SOX regimen followed by camrelizumab plus apatinib |
| 6 | Dose 3 | T4bN3M1 | Distant lymph nodes | MSS | TMB-High | 51.4 | CPS < 1 | Partial response | D2 | Grade 0 | R0 | 0^4^ | None |
| 7 | Dose 3 | T4aN3M1 | Liver, peritoneum | MSS | TMB-Low | 38.2 | Unknown | Partial response | D2 | Grade 1 | R0 | Stage II^5^ | One cycle of SOX regimen plus camrelizumab and apatinib |
| 8 | Dose 3 | T4bN3M1 | Liver, distant lymph nodes | MSS | TMB-Low | 30.2 | Unknown | Partial response | D2 | Grade 2 | R0 | Stage IV | None |
| 9 | Dose 3 | T3N2M1 | Liver, distant lymph nodes | MSS | TMB-Low | 12.2 | CPS < 1 | Partial response | D2 | Grade 1 | R0 | Stage IV | Two cycles of SOX regimen plus camrelizumab and apatinib |
| 10 | Dose 3 | T3N2M1 | Distant lymph nodes | MSS | TMB-High | 25.7 | CPS ≥ 1 | Partial response | D2 | Grade 2 | R0 | Stage IV | Three cycles of SOX regimen plus camrelizumab and apatinib |

^1, 2, 4^ indicated that patients 1, 2, and 6 underwent preoperative evaluation showing a PR, with the disappearance of distant lymph nodes and intraoperative removal of regional lymph nodes. ^3^ indicated that patient 3 had partial removal of liver tissue during surgery, but postoperative pathology revealed no atypical cells in the liver tissue. ^5^ indicated that patient 7, evaluated as PR before surgery with the disappearance of liver metastases, underwent intraoperative removal of peritoneal tissue. However, postoperative pathology showed the absence of atypical cells in the peritoneal tissue. Best overall response was assessed by Response Evaluation Criteria in Solid Tumors (version 1.1). Pathological regression was assessed by tumor regression grade, which was the four-tiered tumor regression grading system recommended by the NCCN guideline.

MSI: Microsatellite instability; MSS: Microsatellite Stable; TMB: Tumor mutational burden; SOD: The sum of longest diameters; CPS: Combined positive score.

**Table S3. Antibodies used in the study**

|  | **Target** | **Source** | **Dilution** | **Opal fluorophores** | **Color** |
| --- | --- | --- | --- | --- | --- |
| **Panel 1** | CD163 | Abcam, ab182422 | 1:500 | Opal 620 | Red |
|  | CD8 | Abcam, ab178089 | 1:200 | Opal 690 | Magenta |
|  | CD68 | Abcam, ab213363 | 1:1000 | Opal 480 | Cyan |
|  | PD-1 | CST, D4W2J, 86163S | 1:200 | Opal 520 | Green |
|  | PD-L1 | CST, E1L3N, 13684S | 1:400 | Opal 570 | Yellow |
| **Panel 2** | CD20 | DAKO, L26, IR604 | 1:1 | Opal 620 | Green |
|  | CD3 | DAKO, A0452 | 1:1 | Opal 690 | Magenta |
|  | CD56 | Abcam, ab75813 | 1:1000 | Opal 480 | Cyan |
|  | CD4 | Abcam, ab133616 | 1:100 | Opal 520 | Red |
|  | FOXP3 | Abcam, ab20034 | 1:100 | Opal 570 | Yellow |
| **Detection in common** | pan-CK | Abcam, ab7753 | 1:100 | Opal 780 | White |
