# Supplementary material for: First-line camrelizumab (a PD-1 inhibitor) plus apatinib (an VEGFR-2 inhibitor) and chemotherapy for advanced gastric cancer (SPACE): a phase 1 study
Source: Signal Transduct Target Ther. 2024 Mar 25;9:73. doi: 10.1038/s41392-024-01773-9 (PMC10963362; doi:10.1038/s41392-024-01773-9)
Supplement: Supplementary file 2 — protocol [file 41392_2024_1773_MOESM2_ESM.docx]

**A Single-arm, Dose Escalation and Expansion Study of Low-dose Apatinib in Combination with Camrelizumab and SOX Regimen as First-line Treatment for Advanced Gastric/Gastroesophageal Junction Adenocarcinoma**

| **Protocol No.:** | **NOAH-GC-003** |
| --- | --- |
| **Version No.:** | **V1.0** |
| **Version Date:** | **01 June 2020** |
| **Principal Investigators:** | **Yongbin Ding, Xiaofeng Chen** |
| **Study Site:** | **Jiangsu Province Hospital** |

**Table of Contents**

[Protocol Signature Page 1](#_Toc126073080)

[Protocol Synopsis 2](#_Toc126073081)

[Study Flow Chart 9](#_Toc126073082)

[List of Abbreviations and Definitions of Terms 12](#_Toc126073083)

[1. Study Background 15](#_Toc126073084)

[1.1 Drug Name and Physicochemical Properties 20](#_Toc126073085)

[1.2 Pharmacological Type and Mechanism of Action 20](#_Toc126073086)

[1.3 Pharmacodynamic Studies 21](#_Toc126073087)

[1.4 Toxicology Studies 21](#_Toc126073088)

[1.5 Pharmacokinetic Studies 22](#_Toc126073089)

[1.6 Clinical Research Progress 22](#_Toc126073181)

[2. Study Objectives and Endpoints 25](#_Toc126073182)

[2.1 Study Objectives 25](#_Toc126073183)

[2.2 Primary Study Endpoints 26](#_Toc126073184)

[2.3 Secondary Study Endpoints 26](#_Toc126073185)

[3. Study Design 27](#_Toc126073186)

[3.1 Description of Study Design 27](#_Toc126073187)

[3.2 Overall Design 27](#_Toc126073188)

[4. Selection and Withdrawal of Patients 31](#_Toc126073197)

[4.1 Inclusion Criteria 31](#_Toc126073198)

[4.2 Exclusion Criteria 32](#_Toc126073199)

[4.3 Discontinuation Criteria 34](#_Toc126073200)

[5. Study Procedures and Data Collection 34](#_Toc126073202)

[5.1 Data Collection Plan 34](#_Toc126073203)

[5.2 Screening Visit 34](#_Toc126073204)

[5.3 Visits during the Treatment 35](#_Toc126073205)

[5.4 Follow-up Visit 35](#_Toc126073206)

[6. Efficacy Evaluation 36](#_Toc126073207)

[6.1 Primary Variables and Observation Methods 36](#_Toc126073208)

[6.2 Secondary Variables and Observation Methods 36](#_Toc126073209)

[7. Safety Evaluation 37](#_Toc126073210)

[7.1 Observation of Adverse Events 37](#_Toc126073211)

[7.2 AE Grading 38](#_Toc126073212)

[7.3 AE Recording 38](#_Toc126073213)

[7.4 Determination of Relationship between AE and Investigational Product 38](#_Toc126073214)

[7.5 Serious Adverse Events 39](#_Toc126073215)

[7.6 Management Comments for Adverse Events 45](#_Toc126073216)

[8. Statistical Methods 50](#_Toc126073217)

[8.1 Statistical Analysis Datasets 50](#_Toc126073218)

[8.2 Statistical Analysis Plan 51](#_Toc126073219)

[9. Quality Control and Quality Assurance 52](#_Toc126073220)

[10. Ethical, Regulatory and Administrative Principles 52](#_Toc126073221)

[10.1 Ethical Principles 52](#_Toc126073222)

[10.2 Laws and Regulations 52](#_Toc126073223)

[10.3 Data Protection 52](#_Toc126073224)

[10.4 Confidentiality Agreement 52](#_Toc126073225)

[10.5 Record Retention 53](#_Toc126073226)

[10.6 Early Study Discontinuation 53](#_Toc126073227)

[10.7 Sponsor Audits and Inspections by Regulatory Authorities 53](#_Toc126073228)

[11. Protocol Amendments 54](#_Toc126073229)

[12. Use of Documentation and Study Results 54](#_Toc126073230)

[12.1 Ownership and Use of Study Data and Study Results 54](#_Toc126073231)

[12.2 Publication 54](#_Toc126073232)

[13. Clinical Study Progress 54](#_Toc126073233)

[Note: The change of the actual duration of study is not a protocol violation. 55](#_Toc126073234)

[14． References 55](#_Toc126073235)

[Annex I Principles for Management of Immune-related Adverse Events 56](#_Toc126073236)

# Protocol Signature Page

I, as a participating doctor/statistician, have read the protocol of this study.

I have fully discussed the objectives of this study and the contents of this protocol with the study director.

I agree to conduct the study in accordance with this protocol, protocol requirements, ethic principles, and under the guidance of Good Clinical Practice (GCP).

I agree that the contents of this protocol will be kept confidential, will not be disclosed to third parties and will only be used for the conduct of this study.

I understand that I will be notified in writing if this study is terminated prematurely or suspended at any time for whatever reason. Similarly, if I decide to withdraw from the conduct of this study, I will immediately notify the leading site and the principal investigators of the study in writing.

Unit:________________

Signature:________________

Date: ________________

# Protocol Synopsis

| **Study Title** | A Single-arm, Dose Escalation and Expansion Study of Low-dose Apatinib in Combination with Camrelizumab and SOX Regimen as First-line Treatment for Advanced Gastric/Gastroesophageal Junction Adenocarcinoma |
| --- | --- |
| **Study No.** | NOAH-GC-003 |
| **Version No.** | 1.0 |
| **Sponsor** | Jiangsu Province Hospital |
| **Study Nature** | Investigator initiated clinical study |
| **Subjects** | Patients with unresectable locally advanced and advanced metastatic gastric or gastroesophageal junction adenocarcinoma |
| **Objectives** | (1) To evaluate the safety, feasibility and efficacy of low-dose apatinib combined with camrelizumab and SOX regimen as first-line treatment in patients with unresectable locally advanced and advanced metastatic gastric/gastroesophageal junction adenocarcinoma.  (2) To observe the genomic, pathological and immune microenvironment changes of tumor-related tissues before and after treatment, investigate the molecular markers related to efficacy to explore the molecular mechanisms affecting the efficacy of combination therapy, and lay the foundation for future large-scale clinical studies. |
| **Number of Planned Enrollment** | A total of 42 |
| **Principal Investigators** | Prof. Yongbin Ding, Prof. Xiaofeng Chen |
| **Study Site** | Jiangsu Province Hospital |
| **Eligibility Criteria** | **Inclusion Criteria:**   1. Subjects aged 18-75 years (inclusive); 2. Understand study procedures and contents, and voluntarily sign written informed consent form; 3. Have histopathologically and/or cytologically confirmed gastric or gastroesophageal junction adenocarcinoma with clinical stage IV, i.e., including advanced metastatic gastric cancer (cTany Nany M1) and unresectable locally advanced gastric cancer (cT4bNanyM0); 4. Have at least 1 measurable lesion per RECIST 1.1 criteria; 5. Have no prior treatment with VEGFR-targeting agents or PD-1/PD-L1 monoclonal antibodies. Those who have relapsed more than 6 months after the end of adjuvant chemotherapy with prior platinum or paclitaxel and fluorouracil regimens and have no current > Grade 2 toxicity per CTCAE 5.0 are allowed to be enrolled. 6. Performance status score (ECOG PS score): 0-1; 7. Expected survival ≥ 3 months; 8. Have good main organ function, i.e., relevant test indicators within 14 days prior to enrollment meet the following requirements: hemoglobin ≥ 90 g/L (no blood transfusion within 14 days); neutrophil count > 1.5 × 10^9^/L; platelet count ≥ 100 × 10^9^/L; total bilirubin ≤ 1.5 × ULN (upper limit of normal); blood alanine aminotransferase (ALT) or aspartate aminotransferase (AST) ≤ 2.5 × ULN; if liver metastases, ALT or AST ≤ 5 × ULN; endogenous creatinine clearance ≥ 60 mL/min (Cockcroft-Gault formula); cardiac Doppler echocardiography assessment: left ventricular ejection fraction (LVEF) ≥ 50%; 9. Thyroid function indicators: thyroid stimulating hormone (TSH) and free thyroxine (FT3/FT4) are within the normal range or mildly abnormal without clinical significance; 10. Weight above 40 kg (including 40 kg), or BMI > 18.5;   **Exclusion Criteria:**   1. Have previous or current other malignancies. However, cured early-stage tumors, i.e., radically treated basal cell carcinoma of the skin, carcinoma in situ of the cervix, stage I lung cancer and stage I colorectal cancer, which are judged by the investigator to have no impact on the patient's life in a short period, may be excluded. 2. Participated in other drug clinical trials within four weeks; 3. Patients with factors that affect oral administration of drugs (such as inability to swallow, chronic diarrhea, intestinal obstruction, etc.); 4. Have a history of bleeding, any Grade 3 or higher bleeding event per CTCAE 5.0 within 4 weeks prior to screening; 5. Patients with known HER2-positive status; 6. Patients with known or history of central nervous system metastases prior to screening. For patients with clinically suspected central nervous system metastases, CT or MRI must be performed within 28 days prior to enrollment to exclude central nervous system metastases; 7. Have hypertension that cannot be well controlled by a single antihypertensive drug therapy (systolic blood pressure > 140 mmHg, diastolic blood pressure > 90 mmHg); have a history of unstable angina; have newly diagnosed angina within 3 months prior to screening or myocardial infarction within 6 months prior to screening; have arrhythmias (including QTcF: ≥ 450 ms in male and ≥ 470 ms in female) requiring long-term use of antiarrhythmic drugs and ≥ New York Heart Association class II cardiac insufficiency; 8. Have long-term unhealed wounds or incompletely healed fractures; 9. Imaging shows that the tumor has invaded important perivascular areas or, as judged by the investigator, the patient's tumor is highly likely to invade important blood vessels and cause fatal major hemorrhage during treatment; 10. Have abnormal coagulation, with bleeding tendency (INR within normal range without anticoagulant 14 days prior to randomization); patients treated with anticoagulants or vitamin K antagonists such as warfarin, heparin, or analogues thereof; low-dose warfarin (1 mg orally once daily) or low-dose aspirin (no more than 100 mg daily) is permitted for prophylactic treatment, provided that international normalized ratio (INR) of prothrombin time ≤ 1.5; 11. Have arterial/venous thromboembolic events, such as cerebrovascular accident (including transient ischemic attack), deep vein thrombosis (except for venous thrombosis caused by venous catheterization in prior chemotherapy that have resolved as judged by the investigator) and pulmonary embolism within 6 months prior to screening; 12. Urinalysis shows urinary protein ≥ ++ and 24-h urinary protein quantitation > 1.0 g; 13. Previous use of immune targeted treatment drugs; 14. Have a history of immunodeficiency, or other acquired, congenital immunodeficiency diseases, or have a history of organ transplantation; 15. Patients with pneumonia, pneumonitis or interstitial pneumonia, and other patients requiring corticosteroids; 16. Have a history of serious chronic autoimmune diseases, such as systemic lupus erythematosus; a history of inflammatory bowel diseases such as ulcerative enteritis and Crohn's disease, and a history of chronic diarrhoeal diseases such as irritable bowel syndrome; a history of sarcoidosis or tuberculosis; a history of active hepatitis B and hepatitis C, and HIV infection. Patients with well-controlled non-serious immune diseases, such as dermatitis, arthritis and psoriasis, are allowed to be enrolled. Patients with hepatitis B virus titer < 500 copies/ml are allowed to be enrolled. 17. Patients with hypersensitivity to human or murine monoclonal antibodies; 18. Patients with a history of anti-psychotics abuse and unable to abstain or a history of mental disorder; 19. Patients with pleural effusion or ascites with clinical symptoms requiring clinical intervention; 20. Patients who do not follow the doctor's advice, do not take drugs according to the requirements, or have incomplete data, which may affect the judgment of efficacy or safety; 21. Patients with concomitant diseases that seriously endanger the patient's safety or affect the patient's completion of the study as judged by the investigator; |
| **Subject Withdrawal/Treatment Discontinuation Criteria** | a. The subject withdraws the informed consent and requests withdrawal;  b. The subject has disease progression (based on investigator assessment and radiographic evidence) or intolerable drug toxicity during treatment;  c. The subject experiences any clinical adverse reaction, laboratory test abnormalities, or intercurrent illnesses, and the investigator believes that continued participation in the study is not in the best interest of the patient;  d. Other conditions in which withdrawal from the study is deemed necessary by the investigator, for instance, the subject losses the ability to express his/her will freely due to confinement or isolation |
| **Regimen** | The study consists of two phases: dose escalation phase (Phase 1a) and dose expansion phase (Phase 1b);  In Phase 1a, every 3 weeks as a treatment cycle, 3 patients will be enrolled in one of the following three treatment regimen groups: 1. apatinib 250 mg, po, qod + camrelizumab 200 mg, i.v., d1 + oxaliplatin 100 mg/m^2^, d1, i.v. + tegafur 40 mg, po, bid, d1-14; 2. apatinib 250 mg, po, qod + carbarelizumab 200 mg, i.v., d1 + oxaliplatin 130 mg/m^2^, d1, i.v., + tegafur 40 mg, po, bid, d1-14; 3. apatinib 250 mg, po, qd + camrelizumab 200 mg, i.v., d1 + oxaliplatin 130 mg/m^2^, d1, i.v. + tegafur 40 mg, po, bid, d1-14. The treatment will continue until unacceptable toxicity, disease progression, or death, or completion of the maximum of eight cycles of SOX regimen and the two-year duration for camrelizumab and apatinib;  In Phase 1b, every 3 weeks as a treatment cycle, the patients will be treated with the most appropriate regimen determined in Phase 1 (RP2D), until unacceptable toxicity, disease progression, or death, or completion of the maximum of eight cycles of SOX regimen and the two-year duration for camrelizumab and apatinib.  (Note: in order to fully exert the immunomodulatory and vascular normalization effects of anti-angiogenic drugs, only during the first cycle, oral apatinib will be given for 7 to 13 days, followed by camrelizumab combined with apatinib and SOX regimen in this study.) |
| **Primary Study Endpoints** | Maximum tolerated dose (MTD) and objective response rate (ORR) |
| **Secondary Study Endpoints** | Progression-free survival (PFS), overall survival (OS), disease control rate (DCR), and duration of response (DOR). |
| **Primary Safety Variables** | Adverse events (AEs), serious adverse events (SAEs), drug-related AEs and SAEs. |
| **Response Assessment Criteria** | Assessed per RECIST 1.1 |
| **Exploratory Variables** | Relationship between gene mutations in tissues and blood at baseline and efficacy; relationship between dynamic cfDNA alterations and efficacy. Relationship between changes in specific T-cell subsets in blood and efficacy, autophagy-related genes (Beclin-1, P53, and Klotho genes and LC3 protein), Pl3K/Akt/mTOR and AMPK/mTOR signaling pathways, tumor microenvironment and host immune system response biomarkers (PD-L1, CD8+ TILs, etc.) and regulatory factors (non-coding RNAs). |
| **Study Duration** | The study is expected to last 30 months, including an 18-month enrollment period.   - First patient enrolled/start of study: 01 June 2020 - Last patient enrolled: 01 December 2021 - End of study: December 2022 (more than 1 year after last eligible patient enrolled) - Database lock date: February 2023 - Report date: February 2023   Note: The change of the actual duration of study is not a protocol violation. |
| **Statistical Description** | The ORR and DCR along with their 95% confidence intervals (CIs) will be calculated using the Clopper-Pearson method. PFS, EFS, and OS curves will be plotted by the Kaplan-Meier method, and their 95% CIs will be calculated by the Brookmeyer-Crowley method. Subgroup analysis of ORR will be performed based on baseline patient characteristics. The survival curves of the different subgroups of patients will be compared using the log-rank test. Categorical variables will be compared using Fisher’s exact test. Comparisons of unpaired continuous variables will be performed using the unpaired Wilcoxon test, and Wilcoxon paired test will be used for paired samples. |

# Study Flow Chart

| **Item** | **Screening** | **C1** | **C2** | **C3** | **C4** | **C5** | **C6** | **Cn** | **90 days post-dose** | **Survival follow-up 1** | **Survival follow-up n** |
| --- | --- | --- | --- | --- | --- | --- | --- | --- | --- | --- | --- |
|  | **V1(-D14-D0)** | **V2** | **V3** | **V4** | | **V5** | | **Vn** | **Vn+1** | **V101** | **Vn01** |
| Signing the ICF | **√** |  |  |  | |  | |  |  |  |  |
| Demographics | **√** |  |  |  | |  | |  |  |  |  |
| Tumor history | **√** |  |  |  | |  | |  |  |  |  |
| Surgical history of primary lesion | **√** |  |  |  | |  | |  |  |  |  |
| Concomitant medical history | **√** |  |  |  | |  | |  |  |  |  |
| Drug allergy history | **√** |  |  |  | |  | |  |  |  |  |
| Tumor markers | **√** | **√** | **√** | **√** | | **√** | | **√** |  |  |  |
| Vital signs | **√** | **√** | **√** | **√** | | **√** | | **√** |  |  |  |
| Physical examination | **√** | **√** | **√** | **√** | | **√** | | **√** |  |  |  |
| Performance status score | **√** |  | **√** | **√** | | **√** | | **√** |  |  |  |
| Hematology | **√** | **√** | **√** | **√** | | **√** | | **√** |  |  |  |
| Blood chemistry | **√** | **√** | **√** | **√** | | **√** | | **√** |  |  |  |
| Urinalysis ^a^ | **√** | **√** | **√** | **√** | | **√** | | **√** |  |  |  |
| Thyroid function | **√** | **√** | **√** | **√** | | **√** | | **√** |  |  |  |
| Coagulation function | **√** | **√** | **√** | **√** | | **√** | | **√** |  |  |  |
| Pregnancy test ^b^ | **√** |  |  |  | |  | |  |  |  |  |
| Electrocardiogram ^c^ | **√** | **√** | **√** | **√** | | **√** | | **√** |  |  |  |
| Quality of life score ^d^ | **√** |  | **√** | **√** | | **√** | | **√** |  |  |  |
| Imaging ^e^ | **√** |  | **√** | **√** | | **√** | | **√** |  |  |  |
| Inclusion criteria | **√** |  |  |  | |  | |  |  |  |  |
| Exclusion criteria | **√** |  |  |  | |  | |  |  |  |  |
| Enrollment assessment | **√** |  |  |  | |  | |  |  |  |  |
| Concomitant medications | **√** |  | **√** | **√** | | **√** | | **√** |  |  |  |
| Additional tests |  |  |  |  | |  | |  |  |  |  |
| Adverse events |  |  | **√** | **√** | | **√** | | **√** | **√** |  |  |
| Serious adverse events |  |  | **√** | **√** | | **√** | | **√** |  |  |  |
| Study summary |  |  |  |  | |  | |  |  |  | **√** |
| Survival follow-up |  |  |  |  | |  | |  |  | **√** | **√** |

Note: all examinations/tests are recommended items, and the specific examinations/tests will be based on clinical practice.

1. Physical status score, physical examination, quality of life score, hematology, urinalysis, stool routine, blood chemistry, coagulation function, pregnancy test and electrocardiogram must be collected within 1 week before administration.

2.a: urinalysis: urinalysis is not required for female patients during the period.

3.b: pregnancy test: for women of childbearing potential.

4.c: electrocardiogram: QTc interval should be recorded for 3 consecutive times, with an interval of approximately 5 minutes; ECGs should be performed before administration at screening, and after administration at each visit thereafter; cardiac color ultrasonography should be supplemented for patients with clinically significant ECG abnormalities; in case of symptoms such as precordial pain and palpitations, myocardial zymograms (creatine kinase and lactate dehydrogenase) should be tested immediately.

5.d: quality of life score: it must be performed before response assessment; every 2 cycles for the first 6 cycles, then every 3 cycles thereafter.

6.e: imaging examination: screening period: including chest CT. CT/MRI of the abdomen plus pelvis is required when abdominal or pelvic metastases are clinically suspected. Brain CT/MRI is required when CNS metastases are clinically suspected. Bone PET-CT is required when bone metastases are clinically suspected. During treatment: the first response assessment will be performed at the end of Cycle 2, and confirmation of response will be performed 8 weeks after the first response assessment; during the first 4 months, evaluations were performed every 6 weeks (±7 days), evaluations were performed every 12 weeks (±7 days) thereafter, and additional scans will be performed as clinically indicated.

7. Follow-up 1 (V101 can be repeated): survival follow-up, with visit numbers as 101, 102, 103, etc.

# List of Abbreviations and Definitions of Terms

| Abbreviations and Terms | Full Text in English |
| --- | --- |
| ADA | Anti-drug antibody |
| ADRs | Adverse drug reactions |
| AE | Adverse event |
| AKP | Alkaline phosphatase |
| ALT | Alanine aminotransferase |
| ANC | Absolute neutrophil count |
| APTT | Activated partial thromboplastin time |
| AST | Aspartate aminotransferase |
| AUC | Area under curve |
| BP | Blood pressure |
| BUN | Blood urea nitrogen |
| CFDA | China Food and Drug Administration |
| Cl | Chlorine |
| Cr | Creatinine |
| CR | Complete response |
| CRF | Case report form |
| CT | Computed tomography |
| CTCAE | Common Terminology Criteria for Adverse Events |
| DBIL | Direct bilirubin |
| DCR | Disease control rate |
| DLT | Dose limited toxicity |
| ECG | Electrocardiogram |
| ECOG | Eastern Cooperative Oncology Group |
| eCRF | Electronic Case report form |
| EDC | Electronic data collection |
| FAS | Full analysis set |

| Abbreviations and Terms | Full Text in English |
| --- | --- |
| FDA | Food and Drug Administration |
| FT3 | free triiodothyronine |
| FT4 | free thyroxine |
| GCP | Good Clinical Practice |
| Glu | Glucose |
| Hb | Hemoglobin |
| IBIL | Indirect bilirubin |
| irAE | Immune-related adverse event |
| INR | International Normalized Ratio |
| IRB | Institutional review board |
| ITT | intend to treat |
| LLN | lower limits of normal |
| mPFS | median progression-free survival |
| MRI | Magnetic Resonance Imaging |
| MTD | Maximum tolerated dose |
| MPR | Major Pathologic Response |
| NCCN | National Comprehensive Cancer Network |
| NCI-CTC | National cancer institute Common Terminology Criteria |
| ORR | Objective response rate |
| OS | Overall survival |
| PD | Progressive disease |
| PD-1/PD-L1 | Programmed death 1/programmed death ligand 1 |
| PFS | Progression-Free-Survival |
| PK | Pharmacokinetics |
| PLT | Platelet |
| PPS | Per-Protocol Set |

| Abbreviations and Terms | Full Text in English |
| --- | --- |
| PR | Partial response |
| PT | Prothrombin time |
| QoL | Quality of life |
| RBC | Red blood cell |
| RO | Receptor occupancy |
| RECIST | Response evaluation criteria in solid tumors |
| SAE | Serious adverse event |
| SD | Stable disease |
| SOP | Standard Operation Procedure |
| SS | Safety Analysis set |
| TB | Total bilirubin |
| TC | Total cholesterol |
| TCM | Traditional Chinese Medicine |
| TG | Triglyceride |
| TP | Plasma total protein |
| TSH | Thyroid-stimulating hormone |
| TT | Thrombin time |
| UA | Blood uric acid |
| UICC | Union for International Cancer Control |
| ULN | Upper limit of normal |
| WBC | White blood cell |

# 1. Study Background

Gastric cancer is the second most common malignant tumor with high morbidity and mortality in China, among which stage IV or unresectable advanced gastric cancer has a very poor prognosis. Previous treatments for advanced gastric cancer were mostly chemotherapy alone, or palliative treatment of chemotherapy combined with targeted drugs. However, recent clinical studies have raised the following challenges to this standard of care: first, checkpoint immunotherapy in combination with chemotherapy and targeted therapy may significantly improve the response rate; second, low-dose anti-angiogenic therapy can lead to vascular normalization, improve local hypoxia and chemotherapy resistance while modulating the immune microenvironment, and further enhance the efficacy of immunotherapy; third, transformation therapy has become a hotspot of current clinical studies, and its meaningful practices show: high completion degree of perioperative chemotherapy and superior clinical benefit compared to postoperative adjuvant chemotherapy; first-line treatment of apatinib in combination with chemotherapy improves the surgical transformation rate in patients with advanced gastric cancer; based on the abundance of primary tumor antigens and the presence of tumor-infiltrating lymphocytes, cross-line or perioperative immunotherapy may be more conducive to the efficacy of immunotherapy. These preliminary exploratory findings are expected to improve the clinical efficacy in patients with advanced gastric cancer, and even effectively control the distant metastasis while down-staging the primary lesion, thereby striving for R0 surgery to improve the prognosis.

Traditionally, advanced gastric cancer refers to pathological stage IV gastric cancer, i.e., generally known metastatic gastric cancer (M1), including non-regional lymph node metastasis (such as para-aortic lymph node metastasis), positive abdominal exfoliated cancer cells (CY1), peritoneal seeding metastasis, and distant organ metastases such as liver metastasis. However, there have also been some changes in the definition of stage IV gastric cancer. In the 8^th^ edition of American Joint Committee on Cancer (AJCC) Cancer Staging Manual, the concept of clinical stage IV gastric cancer was newly proposed, i.e., including clinically judged metastatic gastric cancer (cM1) and tumors invading adjacent organs (cT4b). Because surgery plays an important role in improving the prognosis and quality of life of patients with gastric cancer, the proposal of this clinical staging also reflects the importance of preoperative evaluation and treatment of gastric cancer. Therefore, patients with advanced gastric cancer in a broad scope should include not only pathological stage IV gastric cancer patients, but also include patients with severe local lesions, especially those with invasion of peripheral organs and severe lymph node metastases, who are also the main subjects of transformation therapy in addition to conventional palliative chemoradiotherapy and supportive care. The nature of transformation therapy is to improve the survival rate of patients with advanced tumors who are difficult to perform R0 surgery due to the limitation of surgical technique or biological behavior of tumor. Combined treatments such as active and effective chemotherapy can effectively control the distant metastases while down-staging the primary lesion, and then either debulking or R0 surgery can improve the survival rate of patients with advanced tumors to some extent. However, some tumors with worse biological behavior and higher degree of malignancy, such as aggressive Borrman types III and IV and Lauren's classification as diffuse gastric cancer, usually have poor prognosis, and it is difficult to benefit from surgery, so the patients can only receive palliative and conservative treatment for advanced tumors.

Targeted therapies for gastric cancer have some survival benefits compared to conventional therapies, mainly attributing to anti-angiogenic and anti-HER-2 agents. Human epidermal growth factor receptor 2 (HER-2) is overexpressed in about 7% -34% of gastric cancer and is a biological feature of poor prognosis after gastric cancer surgery. Study TOGA has demonstrated the efficacy of trastuzumab (Herceptin, H) in combination with XP (capecitabine + cisplatin) chemotherapy in advanced gastric cancer, which not only prolongs OS but also improves objective response rates (47% vs 35%) compared with chemotherapy alone. In addition, some small single-arm studies have reported that anti-HER-2 monoclonal antibody has the value of increasing the R0 resection rate and pathological response rate in the perioperative treatment of HER-2-overexpressing locally advanced gastric cancer. In the field of targeted drug therapy for gastric cancer, in addition to trastuzumab, anti-angiogenesis inhibitors ramucirumab and apatinib have successfully completed the phase 2/3 clinical studies. The results of the Phase 3 Study REGARD and RAINBOW suggests that ramucirumab alone or combination treatment has a significant advantage in prolonging survival of patients. Based on these results, FDA approved ramucirumab as second-line treatment for advanced gastric or gastroesophageal junction cancer. As an oral small-molecule TKI of VEGFR2 independently developed in China, apatinib was formally approved for marketing in 2014 for the third or higher line of treatment of advanced gastric cancer. Currently, several institutions are investigating apatinib for the cross-line treatment of gastric cancer. The results may be very promising. In addition, in China, some investigators have attempted to use apatinib in the perioperative treatment of gastric cancer, and the experience of chemotherapy combined with apatinib in the transformation therapy of unresectable advanced gastric cancer is reported for the first time. Thirty-three advanced gastric cancer patients with liver metastasis, peritoneal metastasis or para-aortic lymph node metastasis were treated with PTX + tegafur + apatinib for 2 cycles. Apatinib was given at a dose of 500 mg/d, p.o., then stopped, and a cycle of PTX + tegafur was added. Of the 28 evaluable patients, 21 achieved partial response (PR), 5 had stable disease (SD), and 2 had progressive disease (PD). The overall response rate was 75.0%, and the disease control rate was 92.9%. Of the 21 patients who achieved PR, 18 achieved R0 resection, with treatment-related toxic and side effects within the manageable range. The results of this study preliminarily demonstrate the efficacy of apatinib in combination with chemotherapy as first-line treatment for advanced unresectable gastric cancer, but large randomized controlled studies are lacking.

In recent years, immunotherapy has been regarded as one of the most promising therapeutic strategies in the field of tumor therapy. Especially, checkpoint inhibitors have brought great changes in the treatment mode of advanced malignant tumors. The hotspot of research is antibodies against programmed death-1 (PD-1) and cytotoxic T lymphocyte-associated antigen-4 (CTLA-4). FDA has approved nivolumab, pembrolizumab, atezolizumab, and durvalumab for the treatment of malignant tumors such as lung cancer, colorectal cancer, esophageal cancer, and gastric cancer, but the overall objective response rate of single agent is only 20% to 30%. More and more studies suggest that checkpoint immunotherapy in combination with intravenous chemotherapy may substantially improve the response rate of monotherapy. In Study ATTRACTION-04, patients with advanced gastric cancer were treated with nivolumab in combination with SOX (S-1 + oxaliplatin) or XELOX (xeloda + oxaliplatin) as the first-line regimen, and the objective response rate (ORR) was 57.1% and 76.5% in the SOX and XELOX groups, respectively. With the popularization of PD-1/PD-L1 inhibitors, breakthrough progress has been made in the cross-line application of neoadjuvant therapy for lung cancer and melanoma successively, which has gradually caused a hot spotlight on the use of immune monoclonal antibodies for perioperative treatment of multiple solid tumors. The ongoing Phase 3 Study ATTRACTION-05 in Japan is designed to observe the efficacy of nivolumab in combination with SP or SOX chemotherapy as adjuvant treatment for stage III gastric cancer. The global Phase 3 Study KEYNOTE-585 is designed to evaluate the efficacy of pembrolizumab in combination with 5-FUs in the perioperative treatment of locally advanced resectable gastric cancer. Currently, there is no predictive biomarker to screen the population who can benefit from PD-1 monoclonal antibody in patients with advanced gastric cancer and Study KEYNOTE-062 of first-line combination chemotherapy has a negative result, supporting the prospect of immune checkpoint inhibitors in the perioperative treatment of gastric cancer.

Successful immunotherapy requires not only the infiltration of immune cells, but also the immune-supported microenvironment to maintain the proliferation and function of T cells. Vascular normalization just provides such an environment for immunotherapy. Therefore, more and more attention has been attracted to vascular normalization combined with immunotherapy. At present, studies on their combination therapy have been conducted in a variety of tumor models. A preclinical study and its clinical design based on lung cancer models published in the Cancer Immunology Research in 2019 showed that the use of low-dose apatinib (250 mg/d) reduced tumor hypoxia, increased CD8+ T cell infiltration, and impeded the recruitment of tumor-associated macrophages (TAMs) and myeloid-derived suppressor cells (MDSCs), suggesting an immunomodulatory effect of anti-angiogenic drugs. It was also observed that low-dose apatinib only maintained low immune checkpoint molecular expression for a period of time and PD-L1 level returned to upregulation on Day 14, so it was recommended to initiate anti-PD-1/PD-L1 therapy after 14 days. Another study improved the efficacy of anti-PD-1/PD-L1 therapy by combining low-dose anti-VEGFR2 antibody in breast cancer models, indicating an enhanced effect of vascular normalization on immunotherapy. However, it is still a challenge to successfully combine the two therapies in clinical practice. Firstly, the time window of vascular normalization is difficult to define. Therefore, the search for reliable markers is helpful in combining immunotherapy within the optimal time window for vascular normalization. It has been shown that there is a strong correlation between CD8+ T cell infiltration and vascular normalization in tumor tissue and it is expected to be an effective biomarker of vascular normalization. Secondly, it is also difficult to choose the dose for vascular normalization. Excessive anti-angiogenic therapy also inhibits anti-tumor immunity. At present, low doses of anti-angiogenic drugs are preferred, but the determination of specific doses remains to be further studied. Combined with the above preliminary exploration of these treatment modalities, it is expected to improve the clinical efficacy in patients with advanced gastric cancer, and even effectively control the distant metastasis while down-staging the primary lesion, thereby striving for R0 surgery to improve the prognosis.

Fluorouracil, platinum, and paclitaxel have been mostly used for the treatment of advanced gastric cancer in the past, based on which different drug combinations are applied. However, several studies have shown that the treatment effect in advanced gastric cancer has not significantly improved, and the median survival time of patients remains at 10-14 months. Even with the addition of targeted therapies such as bevacizumab and trastuzumab, there is no significant improvement in prognosis. With the advent of PD-1/PD-L1 inhibitors and their great progress in neoadjuvant treatment of lung cancer and melanoma, preoperative addition of PD-1/PD-L1 inhibitors may provide more options for the transformation therapy of advanced unresectable gastric cancer. Furthermore, appropriate low-dose anti-angiogenic drugs can induce vascular normalization of tumor, reduce the degree of hypoxia, promote the infiltration of CD8+ T lymphocytes and enhance tumor immunotherapy. In turn, the activation of immune cells can promote the vascular normalization, so they form a good positive feedback cycle, which provides a theoretical basis for anti-angiogenic therapy combined with immune checkpoint inhibitors in the treatment of malignant tumors. Although a large number of preclinical studies have shown potential efficacy benefits of immune checkpoint inhibitors in combination with anti-angiogenic agents, it is also critical for some patients to continue to explore the value of both treatments in the transformation therapy of advanced gastric cancer, as well as the optimal sequence and timing of each treatment combination, whether they are administered concomitantly or sequentially.

## 1.1 Drug Name and Physicochemical Properties

[Generic Name] Recombinant Humanized Anti-PD-1 Monoclonal Antibody Injection (Camrelizumab)

[English Name] Camrelizumab for Injection

[Molecular Weight] ~ 143 KD

[Dosage Form] Lyophilized Powder for Injection

[Strength] 200 mg/vial

## 1.2 Pharmacological Type and Mechanism of Action

Programmed death-1 (PD-1) is a protein receptor on the surface of T cells, which participates in the process of cell apoptosis. PD-1 is a member of the CD28 family and is mainly expressed on activated T cells, B cells, and myeloid cells. PD-1 has two ligands, i.e., PD-L1 and PD-L2. PD-L1 is mainly expressed on T cells, B cells, macrophages and dendritic cells, and can be up-regulated on activated cells. Whereas the expression of PD-L2 is relatively limited, it is mainly expressed on antigen-presenting cells, such as activated macrophages and dendritic cells.

The humanized anti-PD1 monoclonal antibody can specifically bind to PD-1 and block the interaction of PD-1 with its ligand, allowing T cells to restore immune responses against tumors.

## 1.3 Pharmacodynamic Studies

The results of our affinity experiments on the binding of the antibody camrelizumab to human, monkey and murine antigens showed that the affinities of the antibody camrelizumab to human and monkey PD1 antigens were 6.9 nM and 4.1 nM, respectively, which were quite close, while there was no measurable binding to murine PD1 antigen. Camrelizumab has an affinity of 3.0 nM for antigen (human PD-1) and its activity is comparable to that of the control antibodies nivolumab and pembrolizumab.

The results of the antibody camrelizumab blocking PD-1/PD-L binding experiments showed comparable *in vitro* blocking activities of camrelizumab and nivolumab/pembrolizumab on PD-1/PD-L1 binding. The blocking activity IC_50_ of the antibodies camrelizumab, nivolumab, and pembrolizumab was 0.70 nM/0.79 nM and 0.79 nM/0.77 nM, respectively.

## 1.4 Toxicology Studies

In the preclinical acute toxicity study in cynomolgus monkeys, 8 cynomolgus monkeys (half males and half females) were randomly divided into 2 groups. Animals in Group 2 were administered camrelizumab via intravenous injection at doses of 200, 400 and 800 mg/kg every other day in a dose escalation manner. There were no camrelizumab-related clinical symptoms or changes in body weight, food consumption and hemagglutination. Decreased lymphocytes were observed in both males and females at doses ≥ 200 mg/kg; increased serum globulin and decreased albumin were observed in both males and females at doses ≥ 400 mg/kg. These changes were not considered to be harmful due to their small magnitude. The maximum tolerated dose (MTD) of camrelizumab is ≥ 800 mg/kg.

In the completed preclinical long-term toxicity study in cynomolgus monkeys, camrelizumab was well tolerated at 20, 50 and 100 mg/kg/dose by intravenous injection once weekly for 4 weeks (5 doses in total) in both sexes. There were no camrelizumab-related clinical symptoms such as injection site irritation, or changes in body weight, food consumption, body temperature, ECG, blood pressure, heart rate and respiratory parameters; there were also no camrelizumab-related changes in B- and T-lymphocyte typing, cytokines, immunoglobulin and complement parameters, and no camrelizumab-related changes in organ weights and macroscopic lesions or histopathological changes.

## 1.5 Pharmacokinetic Studies

**Pharmacokinetics of Camrelizumab**

PK parameters following a single intravenous infusion of camrelizumab to cynomolgus monkeys are presented in Table 1.

**Table 1-1 PK Parameters Following a Single Intravenous Infusion of Camrelizumab at Different Doses in Cynomolgus Monkeys**

| Dose (mg/kg) | Gender | T_1/2_(hr) | T_max_(hr) | C_max_(ug/mL) | AUC_last_(hr*ug/mL) | Vz(mL/kg) | C1(mL/hr/kg) | MRT_last_(hr) |
| --- | --- | --- | --- | --- | --- | --- | --- | --- |
| 1 | Females | 76.06±32.93 | 0.83±0.29 | 31.16±11.25 | 1716.12±453 | 54.09±14.85 | 0.57±0.17 | 80.95±18.58 |
|  | Males | 91.72±25.26 | 0.83±0.29 | 35.96±13.09 | 2359.7±684.0 | 55.15±20.51 | 0.37±0.06 | 102.23±38.56 |
|  | Total | 83.89 ±27.62 | 0.83±0.26 | 33.56±11.23 | 2037.91 ±627 | 54.62±16.02 | 0.47±0.15 | 91.59 ±29.47 |
| 3 | Females | 92.95±22.60 | 0.83±0.29 | 81.09±12.66 | 6896.79±167 | 40.75±12.66 | 0.44±0.11 | 120.92±49.96 |
|  | Males | 113.54±8.26 | 1.67±0.58 | 71.65±10.85 | 6380.24±206 | 47.05±27.05 | 0.47±0.12 | 127.10±59.24 |
|  | Total | 103.25±18.94 | 1.25±0.61 | 76.37 ±11.74 | 6638.51 ±170 | 43.91±19.21 | 0.46±0.11 | 124.01±49.13 |
| 10 | Females | 169.70±33.96 | 2.17±1.76 | 217.46±20.22 | 31357.28±93 | 41.24±24.76 | 0.33±0.1 | 179.68±73.6 |
|  | Males | 128.94±35.93 | 0.67±0.29 | 251.88±6.49 | 26779.98±72 | 30.9±30.2 | 0.31±0.05 | 113.25±44.39 |
|  | Total | 149.32±40.28 | 1.42±1.39 | 234.67±23.15 | 29068.63±78 | 36.07±25.34 | 0.32±0.07 | 146.46±65.42 |

## 1.6 Clinical Research Progress

**Clinical Research Progress of Camrelizumab**

The first Phase 1 clinical study of camrelizumab was conducted in Australia in 2015. Since the approval for clinical study of national class I new drug (No. 2016L01455) was obtained in February 2016, the Phase 1 clinical studies in multiple solid tumors (mainly nasopharyngeal cancer, non-small cell lung cancer, triple negative breast cancer, melanoma, and various digestive system tumors) have been conducted in the Cancer Hospital of the Chinese Academy of Medical Sciences, Sun Yat-sen University Cancer Center, and Peking University Cancer Hospital. The studies were designed to investigate the safety and tolerability of camrelizumab in humans as the primary study objectives, and to investigate the PK characteristics and preliminary efficacy of camrelizumab in humans; the study used the classical 3 + 3 dose escalation method to investigate the safety and tolerability of camrelizumab in humans, and a dose expansion group was set up to investigate the preliminary efficacy and safety of camrelizumab. Preliminary study results showed that camrelizumab was safe and tolerable, and had certain preliminary efficacy against a variety of solid tumors, including non-small cell lung cancer.

To date (data cutoff 31 October 2016), a total of 111 patients with solid tumors have been enrolled in China and abroad, including 87 patients enrolled in Chinese studies. The tolerability observation results showed that camrelizumab at different doses (1 mg/kg, 3 mg/kg, 10 mg/kg and 60 mg, 200 mg, 600 mg fixed dose) was well tolerated in humans, and no dose-limiting toxicity was observed in any dose group during the tolerability observation period, i.e., the maximum tolerated dose (MTD) exceeded 10 mg/kg or 600 mg fixed dose.

In terms of safety, the overall incidence of adverse events (AEs) in Chinese study was 77% (67/87), mainly skin reaction, pyrexia, hypothyroidism, transaminase increased, gastrointestinal reactions, etc., which were mostly related to the mechanism of action of the drug, and were mostly mild and moderate. The symptoms resolved or disappeared after symptomatic treatment or interruption of camrelizumab.

The incidence of camrelizumab-related AEs judged by the investigator was 59.8% (52/87), mostly mild to moderate, as detailed in the following table:

**Incidence of Drug-related AEs in 3 Phase 1 Clinical Studies of Camrelizumab in China (Data Cutoff 31 October 2016)**

| **System Organ Class/Standard Term** | | | | | **Total (N = 67)** |
| --- | --- | --- | --- | --- | --- |
| **Number of subjects with at least one event** | | | | | **67 (77.0%)** |
| **Investigations** | **22 (25.3%)** | **Skin and subcutaneous tissue disorders** | 36(41.4%) | **Gastrointestinal disorders** | 7(8%) |
| Alanine aminotransferase increased | 7 (8.0%) | Hemangioma of skin | 32 (36.8%) | Nausea | 2 (2.3%) |
| Aspartate aminotransferase increased | 7 (8.0%) | Rash | 7 (8.0%) | Diarrhoea | 2 (2.3%) |
| White blood cell count decreased | 3 (3.4%) | Pruritus | 6 (6.9%) | Abdominal distension | 1 (1.1%) |
| Blood bilirubin increased | 3 (3.4%) | Rash maculo-papular | 3 (3.4%) | Mouth ulceration | 1 (1.1%) |
| Electrocardiogram QT interval prolongation | 2 (2.3%) | Flushed skin | 1 (1.1%) | Vomiting | 1 (1.1%) |
| Blood creatine kinase isoenzyme increased | 2 (2.3%) | Palmar-plantar erythrodysaesthesia syndrome | 1 (1.1%) | Constipation | 1 (1.1%) |
| Blood troponin increased | 1 (1.1%) | Urticaria | 1 (1.1%) |  |  |
| White blood cell count increased | 1 (1.1%) |  |  | **Blood and lymphatic system disorders** | **4 (4.6%)** |
| Neutrophil count decreased | 1 (1.1%) | **General disorders and administration site conditions** | **18 (20.7%)** | Anaemia | 4 (4.6%) |
| Blood thyroid stimulating hormone | 1 (1.1%) | Pyrexia | 9 (10.3%) |  |  |
| Blood prolactin increased | 1 (1.1%) | Asthenia | 5 (5.7%) | **Nervous system disorders** | **3 ( 3.4%)** |
| Myoglobin blood increased | 1 (1.1%) | Fatigue | 4 (4.6%) | Insomnia | 1 (1.1%) |
| Blood creatine phosphokinase increased | 1 (1.1%) | Chills | 1 (1.1%) | Headache | 1 (1.1%) |
| Blood creatinine increased | 1 (1.1%) |  |  | Dizziness | 1 (1.1%) |
| Hypokalaemia | 1 (1.1%) | Endocrine disorders | **9 (10.3%)** |  |  |
|  |  | Hypothyroidism | 8 (9.2%) | **Hepatobiliary disorders** | **2 ( 2.3%)** |
|  |  | Hyperthyroidism | 1 (1.1%) | Hepatic function abnormal | 2 ( 2.3%) |

The incidence of Grade 3 or higher AEs was 10.3%, and the incidence of Grade 3 or higher drug-related AEs was 2.3%. Grade 3 or higher AEs are shown in the following table:

**Incidence of Grade 3 or Higher AEs in 3 Phase 1 Clinical Studies of Camrelizumab in China (Data Cutoff 31 October 2016)**

| **System Organ Class/Standard Term**  **Number of subjects with at least one event** | | | Total (N = 67)  **67 (77.0%)** |
| --- | --- | --- | --- |
| **Drug-related AEs judged by the investigator are highlighted** | | | |
| **Investigations** |  | **Gastrointestinal disorders** |  |
| Hypochloraemia | 2 (2.3%) | Abdominal pain | 1 (1.1%) |
| Hyponatraemia | 2 (2.3%) | Upper gastrointestinal haemorrhage | 1 (1.1%) |
| Aspartate aminotransferase increased | 2 (2.3%) |  |  |
| Blood bilirubin increased | 1 (1.1%) | **Metabolism and nutrition disorders** | **1 (1.1%)** |
| Hypophosphatemia | 1 (1.1%) | Hypercalcaemia | 1(1.1%) |
| Alanine aminotransferase increased | 1 (1.1%) |  |  |
| Creatine kinase isoenzyme increased | 1 (1.1%) | Hepatobiliary disorders | **1 (1.1%)** |
|  |  | Hepatic function abnormal | 1 (1.1%) |
| **General disorders and administration site conditions** | **3 (3.4%)** |  |  |
| Pyrexia | 1 (1.1%) | **Musculoskeletal and connective tissue disorders** | 1 (1.1%) |
| PD | 1 (1.1%) | Back pain | 1 (1.1%) |
| Death | 1 (1.1%) | Lumbago | 1 (1.1%) |
| **Blood and lymphatic system disorders** | **2 (2.3%)** | **Endocrine disorders** | 1 (1.1%) |
| Anaemia | 2 (2.3%) | Hypothyroidism | 1 (1.1%) |

Preliminary safety data indicate that the overall safety of camrelizumab is acceptable.

Test of PD1 receptor occupancy showed that a single dose of camrelizumab at 1 mg/kg resulted in more than 80% PD1 receptor occupancy, and the receptor binding of camrelizumab was sustained. It suggested that the administration of clinical doses of camrelizumab could achieve sustained and high PD1 binding and play a role in overcoming immune escape.

The PK study results of camrelizumab in patients with advanced solid tumors showed that the *in vivo* exposure (AUC) and C_max_ of camrelizumab increased proportionally with the administered dose, and no PK saturation was observed. The clearance half-life (t_1/2_) of camrelizumab in humans was about 3.5 days (1-3 mg/kg), and the t_1/2_ at the 200 mg fixed dose was 5.83 ± 0.91 days, supporting q2w (14 days/dose) administration.

In terms of efficacy, as of 15 November 2016, in clinical studies in China, camrelizumab showed preliminary efficacy in solid tumors after failure of multiple standard therapies. The overall objective response rate (ORR) was 20.8% (10/48); the disease control rate (DCR) was 41.7% (20/48). Of these, the objective response rate (ORR) was 9.1% (1/11) and the disease control rate (DCR) was 45.5% (5/11) in patients with non-small cell lung cancer. In addition, similar to the efficacy data reported for similar drugs of the same class, the duration of response/benefit produced by camrelizumab was longer. The duration of response/benefit was longer in patients with objective response or stable disease (as the study is still ongoing and there is no available conclusive data to support it, it is an observational trend).

Camrelizumab in combination with apatinib may improve the short-term objective response rate while preserving the durability of response to immunotherapy. In previous animal studies, the combination regimen of camrelizumab and apatinib showed synergistic anti-tumor efficacy. In both subcutaneous lung adenocarcinoma xenograft models in mice and subcutaneous lung adenocarcinoma metastatic tumor models in mice, low-dose apatinib combined with camrelizumab effectively reduced tumor size, prolonged survival time, and improved survival rate. In addition, the results of the Phase 1b/2 study (NCT03083041) of camrelizumab in combination with apatinib in the treatment of non-small cell lung cancer showed: in the Phase 1b study (n = 27), the ORR was 41.2%, the DCR was 94.1%, the median PFS was 24 weeks in the apatinib 250 mg group, and the apatinib 375 mg group was still under follow-up, with good overall tolerability; in the Phase 2 study, the ORR and DCR were 29.7% and 81.3% in the 91 evaluable patients, respectively, while the ORR was 50% (19/38) in patients with high blood tumor mutation burden (bTMB).

The Phase 1 clinical study of camrelizumab in patients with advanced solid tumors showed that camrelizumab had excellent safety and tolerability; PK and PD characteristics as well as preliminary efficacy worth exploring.

# 2. Study Objectives and Endpoints

## 2.1 Study Objectives

(1) To evaluate the safety, feasibility and efficacy of low-dose apatinib combined with camrelizumab and SOX regimen as first-line treatment in patients with unresectable locally advanced and advanced metastatic gastric/gastroesophageal junction adenocarcinoma.

(2) To observe the genomic, pathological and immune microenvironment changes of tumor-related tissues before and after treatment, investigate the molecular markers related to efficacy to explore the molecular mechanisms affecting the efficacy of combination therapy, and lay the foundation for future large-scale clinical studies.

## 2.2 Primary Study Endpoints

- Phase 1a: Maximum Tolerated Dose (MTD)

The patients will receive the combination regimen of camrelizumab in combination with apatinib and SOX after enrollment in the dose escalation phase. If ≤ 1 patient in each treatment group experiences a dose-limiting toxicity (DLT) within the first 21 days of treatment, the patients will be enrolled in the higher dose combination regimen group for treatment. If ≥ 2 patients experiences a DLT, the dose of the previous combination regimen will be considered as the MTD.

Once the MTD is determined, other patients will be enrolled from this level in the expansion cohort (Phase 1b, Part 2), where intra-patient dose modifications or interruptions of apatinib are allowed. All patients will continue the combination therapy until disease progression, intolerable toxicity, death, or discontinuation for any reason.

A DLT is defined as any Grade 4 hematologic toxicity or any Grade 3 or higher non-hematologic toxicity during the first 28 days of treatment, or any toxicity of camrelizumab or apatinib that leads to a dose delay of ≥ 21 days.

- Phase 1a and 1b: Objective Response Rate (ORR)

It refers to the proportion of patients whose tumor shrinkage reaches partial response (PR) and complete response (CR) for a certain time. Subjects with CR and PR need to be confirmed at the next imaging assessment. Objective response will be assessed using RECIST 1.1 criteria. Patients must have measurable tumor lesions at baseline, and the response evaluation criteria are classified as CR, PR, stable disease (SD), and progressive disease (PD) according to RECIST 1.1 criteria.

## 2.3 Secondary Study Endpoints

- Overall Survival (OS)

Overall survival is defined as the time from the first dose to the patient death due to any cause.

- Progression-free Survival (PFS)

PFS1 was the time from the first dose to the first occurrence of disease progression or death due to any cause. PFS2 was the time from the first dose to the first occurrence of disease progression, disease recurrence, or death due to any cause.

- Disease Control Rate (DCR):

It refers to the percentage of subjects with confirmed complete response, partial response, and stable disease.

- Duration of Response (DOR）

It refers to the time from the first assessment of CR or PR to the first assessment of PD or death due to any reason.

- Drug Safety

Any AEs, including abnormal clinical symptoms and vital signs and laboratory abnormalities, are observed during the clinical study in all patients, and recorded with their clinical characteristics, severity, onset time, duration, treatment methods and prognosis, and correlation with the investigational products. The safety of the drug will be evaluated according to NCI-CTCAE version 5.0 criteria.

# 3. Study Design

## 3.1 Description of Study Design

This is a single-arm, dose-escalation and expansion study to observe and evaluate the safety, feasibility, and efficacy of low-dose apatinib combined with camrelizumab and SOX regimen as first-line treatment in unresectable locally advanced and advanced metastatic gastric/gastroesophageal junction adenocarcinoma, aiming to provide a more optimized treatment strategy for such patients. In addition, this study is designed to observe the genomic, pathological and immune microenvironment changes of tumor-related tissues before and after treatment, investigate the molecular markers related to efficacy to explore the molecular mechanisms affecting the efficacy of combination therapy, and lay the foundation for future large-scale clinical studies.

## 3.2 Overall Design

### 3.2.1 Sample Size

In the Phase 3 randomized controlled study (keynote 062), the objective response rate of chemotherapy (cisplatin + 5-FU) in combination with immunotherapy (pembrolizumab) was 48.6% in patients with CPS > 1 and 52.5% in patients with CPS > 10. Assuming an objective response rate of 50% for chemotherapy in combination with immunotherapy as a basis, the combination with apatinib increases the objective response rate up to 70%. With one-sided α = 0.05, β = 0.2, the calculated sample size is 37 patients. Considering a dropout rate of 10%, 42 samples will be required.

### 3.2.2 Dosing Regimen

The study consists of two phases: dose escalation phase (Phase 1a) and dose expansion phase (Phase 1b);

In Phase 1a, every 3 weeks as a treatment cycle, 3 patients will be enrolled in one of the following three treatment regimen groups: 1. apatinib 250 mg, po, qod + camrelizumab 200 mg, i.v., d1 + oxaliplatin 100 mg/m^2^, d1, i.v. + tegafur 40 mg, po, bid, d1-14; 2. apatinib 250 mg, po, qod + carbarelizumab 200 mg, i.v., d1 + oxaliplatin 130 mg/m^2^, d1, i.v., + tegafur 40 mg, po, bid, d1-14; 3. apatinib 250 mg, po, qd + camrelizumab 200 mg, i.v., d1 + oxaliplatin 130 mg/m^2^, d1, i.v. + tegafur 40 mg, po, bid, d1-14. The treatment will continue until unacceptable toxicity, disease progression, or death;

In Phase 1b, every 3 weeks as a treatment cycle, the patients will be treated with the most appropriate regimen determined in Phase 1 (RP2D), until unacceptable toxicity, disease progression, or death.

(Note: in order to fully exert the immunomodulatory and vascular normalization effects of anti-angiogenic drugs, only during the first cycle, oral apatinib will be given for 7 to 13 days, followed by camrelizumab combined with apatinib and SOX regimen in this study.)

### 3.2.3 Dose Interruption

The investigational products may be interrupted and discontinued based on the toxic and side effects of the investigational products; during the study, camrelizumab is only allowed to be interrupted, with a maximum of 8 weeks of dose interruption; if the dose of camrelizumab is delayed for more than 3 days after the scheduled dose time, the delayed dose will not be made up, and the dose of 200 mg will be continued at the next scheduled dose time. During the expansion phase, the patients will be allowed to have apatinib dose modification or interruption.

If the patient experiences fever (> 38°C, requiring corrective drug treatment), obvious symptoms of gasping, polypnoea, breathlessness or rash during the study, camrelizumab will not be administered at the current or next scheduled dose time prior to symptom resolution. After the symptoms resolve and stabilize for more than 7 days, camrelizumab should be administered at the subsequent scheduled dose time. For patients with fever and gasping, pneumonia must be ruled out by imaging examination before administration as appropriate.

If Grade 4 diarrhoea/colitis, ≥ Grade 3 bilirubin increased, ≥ Grade 3 injection reaction, pneumonia, nephritis, or renal failure, or other Grade 4 drug-related non-hematological toxicities occur during the study, the patient will be discontinued from the investigational product (camrelizumab).

### 3.2.4 Concomitant Medications

**3.2.4.1 Medications Used with Caution or Prohibited during the Study**

Patients should avoid concomitant immunosuppressant therapy such as thymalfasin, interferon, interleukin-2, and other immunosuppressive agents (except for managing drug-related AEs) during the treatment period of this study. Systemic corticosteroids > 10 mg/day (prednisone or equivalents) are prohibited, except for the treatment or control of drug-related AEs occurring during treatment or short-term use as prophylactic therapy. Live vaccines are not allowed within 4 weeks prior to the first dose of investigational product and 60 days after the last dose of investigational product.

**3.2.4.2 Medications and Treatments That May Be Used as Appropriate during the Study**

Palliative and supportive treatments for underlying diseases and symptom management are allowed during the study for concern about the subject's health. Palliative and supportive treatments for disease-related symptoms will depend on the judgment of the investigator and relevant guidelines.

Patients may be treated with bisphosphonates for bone metastases. If pain in bone metastases cannot be effectively controlled by systemic therapy or local analgesia, palliative small-area radiotherapy (radiotherapy area must be < 5% of the bone marrow area) is allowed.

Palliative treatment for lesions beyond the lungs and liver (when patients need treatment to relieve the symptoms due to PD) is allowed during the study, including treatment for pleural effusion, ascites, and pericardial effusion, radiotherapy for craniocerebral lesions, etc.

All concomitant treatments and drugs used within 30 days prior to the start of dosing and during the study should be recorded in the eCRF in strict accordance with GCP. In case of adverse reactions, the patients should be closely observed, active symptomatic treatment should be given if necessary, and the medications used should be recorded and explained on the CRF form. Once a patient discontinues the study treatment, only concomitant medications and concomitant treatments used for AEs occurring during the safety follow-up period should be recorded.

### 3.2.5 Efficacy Evaluation and Analysis

Response assessment is recommended every 6 weeks (± 7 days) in the first 4 months of treatment (based on calendar days and not affected by discontinuation), then every 12 weeks (± 7 days) thereafter and within ± 7 days of the end of the cycle (except for legal holidays). CT or MRI should be performed for evaluation. The imaging technique used for the same patient should be the same at different evaluations, and all imaging data should be retained. Patients who achieve CR, PR, and SD should be confirmed by reexamination 4 weeks after the first evaluation.

Patients who experience PD may continue the study treatment with the consent of the patient if the investigator judges that the patient will continue benefiting from the treatment, and re-assessment will be conducted in 4 weeks; otherwise, the patient will discontinue the study treatment and enter the survival follow-up period.

For patients who withdraw from the study due to intolerable toxicities, if no response assessment is performed within 4 weeks prior to withdrawal, the efficacy evaluation should be performed at the time of withdrawal.

### 3.2.6 Follow-up Within 90 Days After Drug Withdrawal

All patients should continue to be evaluated for safety within 90 days of the end of last dose. AEs, concomitant medications, and concomitant therapies should be assessed at the follow-up visit on Day 90 (± 3 days, except for legal holidays) after the end of treatment.

### 3.2.7 Safety Follow-up

The safety follow-up visit will be performed once a month (± 7 days) for three consecutive times: the first must be performed at the study site, while the 2^nd^ and 3^rd^ visits can be performed by telephone in order to track the AE outcomes.

### 3.2.8 Survival Follow-up

After PD, the subjects will enter the survival follow-up period and be followed up by telephone every 3 months (± 14 days) to collect the survival status (date and cause of death) and other information after the end of study treatment (including received treatment) until the endpoint of death or the subject lost to follow-up.

# 4. Selection and Withdrawal of Patients

## 4.1 Inclusion Criteria

1. Subjects aged 18-75 years (inclusive);
2. Understand study procedures and contents, and voluntarily sign written informed consent form;
3. Have histopathologically and/or cytologically confirmed gastric or gastroesophageal junction adenocarcinoma with clinical stage IV, i.e., including advanced metastatic gastric cancer (cTany Nany M1) and unresectable locally advanced gastric cancer (cT4bNanyM0)；
4. Have at least 1 measurable lesion per RECIST 1.1 criteria;
5. Have no prior treatment with VEGFR-targeting agents or PD-1/PD-L1 monoclonal antibodies. Those who have relapsed more than 6 months after the end of adjuvant chemotherapy with prior platinum or paclitaxel and fluorouracil regimens and have no current > Grade 2 toxicity per CTCAE 5.0 are allowed to be enrolled.
6. Performance status score (ECOG PS score): 0-1;
7. Expected survival ≥ 3 months;
8. Have good main organ function, i.e., relevant test indicators within 14 days prior to enrollment meet the following requirements: hemoglobin ≥ 90 g/L (no blood transfusion within 14 days); neutrophil count > 1.5 × 10^9^ /L; platelet count ≥ 100 × 10^9^ /L; total bilirubin ≤ 1.5 × ULN (upper limit of normal); blood alanine aminotransferase (ALT) or aspartate aminotransferase (AST) ≤ 2.5 × ULN; if liver metastases, ALT or AST ≤ 5 × ULN; endogenous creatinine clearance ≥ 60 mL/min (Cockcroft-Gault formula); cardiac Doppler echocardiography assessment: left ventricular ejection fraction (LVEF) ≥ 50%;
9. Thyroid function indicators: thyroid stimulating hormone (TSH) and free thyroxine (FT3/FT4) are within the normal range or mildly abnormal without clinical significance;
10. Weight above 40 kg (including 40 kg), or BMI > 18.5;

## 4.2 Exclusion Criteria

1. Have previous or current other malignancies. However, cured early-stage tumors, i.e., radically treated basal cell carcinoma of the skin, carcinoma in situ of the cervix, stage I lung cancer and stage I colorectal cancer, which are judged by the investigator to have no impact on the patient's life in a short period, may be excluded.
2. Participated in other drug clinical trials within four weeks;
3. Patients with factors that affect oral administration of drugs (such as inability to swallow, chronic diarrhea, intestinal obstruction, etc.);
4. Have a history of bleeding, any Grade 3 or higher bleeding event per CTCAE 5.0 within 4 weeks prior to screening;
5. Patients with known HER2-positive status;
6. Patients with known or history of central nervous system metastases prior to screening. For patients with clinically suspected central nervous system metastases, CT or MRI must be performed within 28 days prior to enrollment to exclude central nervous system metastases;
7. Have hypertension that cannot be well controlled by a single antihypertensive drug therapy (systolic blood pressure > 140 mmHg, diastolic blood pressure > 90 mmHg); have a history of unstable angina; have newly diagnosed angina within 3 months prior to screening or myocardial infarction within 6 months prior to screening; have arrhythmias (including QTcF: ≥ 450 ms in male and ≥ 470 ms in female) requiring long-term use of antiarrhythmic drugs and ≥ New York Heart Association class II cardiac insufficiency;
8. Have long-term unhealed wounds or incompletely healed fractures;
9. Imaging shows that the tumor has invaded important perivascular areas or, as judged by the investigator, the patient's tumor is highly likely to invade important blood vessels and cause fatal major hemorrhage during treatment;
10. Have abnormal coagulation, with bleeding tendency (INR within normal range without anticoagulant 14 days prior to randomization); patients treated with anticoagulants or vitamin K antagonists such as warfarin, heparin, or analogues thereof; low-dose warfarin (1 mg orally once daily) or low-dose aspirin (no more than 100 mg daily) is permitted for prophylactic treatment, provided that international normalized ratio (INR) of prothrombin time ≤ 1.5;
11. Have arterial/venous thromboembolic events, such as cerebrovascular accident (including transient ischemic attack), deep vein thrombosis (except for venous thrombosis caused by venous catheterization in prior chemotherapy that have resolved as judged by the investigator) and pulmonary embolism within 6 months prior to screening;
12. Urinalysis shows urinary protein ≥ ++ and 24-h urinary protein quantitation > 1.0 g;
13. Previous use of immune targeted treatment drugs;
14. Have a history of immunodeficiency, or other acquired, congenital immunodeficiency diseases, or have a history of organ transplantation;
15. Patients with pneumonia, pneumonitis or interstitial pneumonia, and other patients requiring corticosteroids;
16. Have a history of serious chronic autoimmune diseases, such as systemic lupus erythematosus; a history of inflammatory bowel diseases such as ulcerative enteritis and Crohn's disease, and a history of chronic diarrhoeal diseases such as irritable bowel syndrome; a history of sarcoidosis or tuberculosis; a history of active hepatitis B and hepatitis C, and HIV infection. Patients with well-controlled non-serious immune diseases, such as dermatitis, arthritis and psoriasis, are allowed to be enrolled. Patients with hepatitis B virus titer < 500 copies/ml are allowed to be enrolled.
17. Patients with hypersensitivity to human or murine monoclonal antibodies;
18. Patients with a history of anti-psychotics abuse and unable to abstain or a history of mental disorder;
19. Patients with pleural effusion or ascites with clinical symptoms requiring clinical intervention;
20. Patients who do not follow the doctor's advice, do not take drugs according to the requirements, or have incomplete data, which may affect the judgment of efficacy or safety;
21. Patients with concomitant diseases that seriously endanger the patient's safety or affect the patient's completion of the study as judged by the investigator;

## 4.3 Discontinuation Criteria

1. The subject withdraws the informed consent and requests withdrawal;
2. The subject has disease progression (based on investigator assessment and radiographic evidence) or intolerable drug toxicity during treatment;
3. The subject experiences any clinical adverse reaction, laboratory test abnormalities, or intercurrent illnesses, and the investigator believes that continued participation in the study is not in the best interest of the patient;
4. Other conditions in which discontinuation from the treatment is deemed necessary by the investigator, for instance, the subject losses the ability to express his/her will freely due to confinement or isolation

# 5. Study Procedures and Data Collection

## 5.1 Data Collection Plan

The duration of safety data collection is from the first dose to 90 days after the end of last dose. The severity of AEs will be evaluated according to NCI CTCAE 5.0 criteria.

All AEs will be recorded in the CRF from the first dose to the end of study.

Each patient will have scheduled visits and specific data will be recorded at different time points of the visit.

## 5.2 Screening Visit

**All examinations/tests are recommended items, and the specific examinations/tests will be based on clinical practice.**

The screening visit should be performed within 2 weeks prior to treatment in the first cycle. The following data will be collected within 14 days prior to treatment: medical history (including prior treatment history), vital signs, physical examination, ECOG PS score, hematology (white blood cells (WBC)), neutrophils (ANC), urinalysis, stool routine, liver and kidney functions (total bilirubin (TBIL), conjugated bilirubin (DBIL), ALT, AST, alkaline phosphatase (AKP), total protein (TP), albumin (ALB), urea nitrogen (BUN), creatinine (Cr), γ-glutamyltransferase (γ-GT), lactate dehydrogenase (LDH), electrolytes (K), sodium (Na), chloride (Cl), calcium (Ca), and phosphorus (P)), thyroid function (TSH, T3, T4, FT3, and FT4), coagulation function (PT, APTT, thrombin time (TT), and fibrinogen (Fbg)), HIV/HBV/HCV virus test, ECG, and chest CT (abdominal + pelvic CT/MRI is required for clinical suspicion of abdominal or pelvic metastases; brain CT/MRI is required for clinical suspicion of central nervous system metastases; bone ECT is required for clinical suspicion of bone metastases). Quality of life scoring and pregnancy test (if clinically indicated possible pregnancy) will be performed.

## 5.3 Visits during the Treatment

Vital signs, ECOG PS score, physical examination, hematology, urinalysis, liver and kidney functions, electrolytes, thyroid function (TSH, T3, T4, FT3, and FT4) and coagulation function should be examined and tested before treatment in each cycle; if symptoms such as precordial pain and palpitations occur, myocardial zymogram (creatine kinase and lactate dehydrogenase) should be tested immediately, ECG should be examined at any time, and cardiac color ultrasonography should also be performed. Response assessment will be performed every 6 weeks (± 7 days) in the first 4 months and every 12 weeks (± 7 days) thereafter, regardless of treatment delay, until disease progression, withdrawal of informed consent, or death. When PD is suspected (e.g., symptom deterioration) or a subject withdraws from treatment (if no assessment is performed within 4 weeks), imaging evaluation should also be performed. When clinically indicated, the frequency of monitoring can be increased. The imaging technique used for the same patient should be the same at different evaluations, and all imaging data should be retained.

## 5.4 Follow-up Visit

Patients will enter the post-treatment follow-up period after discontinuation of investigational product. Patients who withdraw due to PD should be followed for survival until the endpoint of death or the subject is lost to follow-up or discontinue from the study.

The following parameters should be recorded during the follow-up period: time to disease progression or death; other tumor therapies; SAEs occurring during the study; and survival (follow-up records by telephone should be maintained).

# 6. Efficacy Evaluation

## 6.1 Primary Variables and Observation Methods

- Maximum Tolerated Dose (MTD)

The patients will receive the combination regimen of camrelizumab in combination with apatinib and SOX after enrollment in the dose escalation phase. If ≤ 1 patient in each treatment group experiences a dose-limiting toxicity (DLT) within the first 21 days of treatment, the patients will be enrolled in the higher dose combination regimen group for treatment. If ≥ 2 patients experiences a DLT, the dose of the previous combination regimen will be considered as the MTD.

Once the MTD is determined, other patients will be enrolled from this level in the expansion cohort (Phase 1b, Part 2), where intra-patient dose modifications or interruptions of apatinib are allowed. All patients will continue the combination therapy until disease progression, intolerable toxicity, death, or discontinuation for any reason. A DLT is defined as any Grade 4 hematologic toxicity or any Grade 3 or higher non-hematologic toxicity during the first 21 days of treatment, or any toxicity of camrelizumab or apatinib that leads to a dose delay of ≥ 21 days.

- Objective Response Rate (ORR)

It refers to the proportion of patients whose tumor shrinkage reaches PR and CR for a certain time. Subjects with CR and PR need to be confirmed at the next imaging assessment. Objective response will be assessed using RECIST 1.1 criteria. Patients must have measurable tumor lesions at baseline, and the response evaluation criteria are classified as complete response (CR), partial response (PR), stable disease (SD), and progressive disease (PD) according to RECIST 1.1 criteria.

## 6.2 Secondary Variables and Observation Methods

- Overall Survival (OS)

Overall survival is defined as the time from the first dose to the patient death due to any cause.

- Progression-free Survival (PFS)

PFS1 was the time from the first dose to the first occurrence of disease progression or death due to any cause. PFS2 was the time from the dose to the first occurrence of disease progression, disease recurrence, or death due to any cause.

- Disease Control Rate (DCR):

It refers to the percentage of subjects with confirmed complete response, partial response, and stable disease.

- Duration of Response (DOR）

It refers to the time from the first assessment of CR or PR to the first assessment of PD or death due to any reason.

- Drug Safety

Any AEs, including abnormal clinical symptoms and vital signs and laboratory abnormalities, are observed during the clinical study in all patients, and recorded with their clinical characteristics, severity, onset time, duration, treatment methods and prognosis, and correlation with the investigational products. The safety of the drug will be evaluated according to NCI-CTCAE version 5.0 criteria.

# 7. Safety Evaluation

## 7.1 Observation of Adverse Events

Adverse event (AE): any untoward medical occurrence in a patient or clinical study subject after receiving a drug or treatment regimen, which does not necessarily have a causal relationship with the treatment.

An AE can therefore be any unfavourable and unintended sign (including an abnormal laboratory finding, for example), symptom, or disease temporally associated with the use of a medicinal product, whether or not considered related to the medicinal product.

Events that occur during the pre- and post-treatment phase are also considered AEs according to regulations. Therefore, the AE or SAE reporting period for safety surveillance begins when the patient is included in the study (signing ICF) and continues until the end of the safety follow-up period.

## 7.2 AE Grading

AEs will be graded into 0-5 according to NCI Common Acute and Subacute Toxicity Grading Criteria (NCI-CTCAE 5.0). AEs not listed in the NCI toxicity grading criteria can be evaluated according to the following criteria:

Grade 1: Mild, asymptomatic or mild; clinical or diagnostic observations only; intervention not indicated;

Grade 2: Moderate; smaller, local or non-invasive treatment is required; or age-related instrumental activities of daily living are limited;

Grade 3: Severe or medically significant but not immediately life-threatening; hospitalization or prolongation of hospitalization indicated; disabling; limiting self care ADL;

Grade 4: Life-threatening; urgent intervention indicated;

Grade 5: Death related to AE.

## 7.3 AE Recording

The name, severity, onset date, duration, action taken, and outcome of AEs occurring during the study will be recorded in detail and truthfully filled in the CRF. Abnormal laboratory data will be recorded in the CRF, and such lab test will be repeated at least weekly until normalization or end of the study.

AEs occurring during the safety follow-up period should be reported and recorded.

## 7.4 Determination of Relationship between AE and Investigational Product

The possible relationship between the AE and the investigational product should be assessed to be "definitely related, probably related, possibly related, unlikely related, not related" (see Table 7-1). The first three levels represent that the AE is related to the investigational product, the total number of subjects with these three levels will be used as the numerator, and the total number of subjects used to evaluate safety will be used as the denominator to calculate the incidence of AE.

Table 7-1 Criteria for Determination of Relationship between AE and Investigational Product

| Criteria | Definitely related | Probably related | Possibly related | Unlikely related | Not related |
| --- | --- | --- | --- | --- | --- |
| Reasonable temporal sequence | Yes | Yes | Yes | Yes | No |
| Known type of drug reaction | Yes | Yes | Yes | No | No |
| Improvement after dechallenge | Yes | Yes | Yes or No | Yes or No | No |
| Recurrence on re-challenge | Yes | ？ | ？ | ？ | No |
| Reaction explained otherwise | No | No | No | Yes | Yes |

## 7.5 Serious Adverse Events

1. Definition of Serious Adverse Events

Serious adverse event (SAE): any medical event that occurs in the course of the clinical trial leading to inpatient hospitalization or prolongation of existing hospitalization, disability, compromised working capacity, life-threatening condition, death, or congenital deformity. An SAE is any unexpected medical occurrence that:

- results in death;
- is life-threatening (refers to an event in which the patient is at risk of death at the time of the event);
- requires inpatient hospitalization or prolongation of existing hospitalization;
- results in persistent or significant disability/incapacity;
- results in congenital anomalies or birth defects;
- is an overdose.

1. Pregnancy

Pregnancies occurring during clinical the study should be reported as SAEs.

1. Disease Progression

Disease progression (including symptoms and signs of progression) should not be reported as an SAE, but death due to disease progression during the study or safety reporting period. Hospitalization for symptoms and signs of disease progression should not be reported as an SAE. During the study or safety reporting period, if the final outcome of cancer is death, the event leading to death must be reported as an SAE.

1. Other Anti-tumor Therapies

For patients who start to receive another anti-tumor therapy, non-fatal AEs should be reported until the start of new anti-tumor therapy. Death must be reported if it occurs within the reporting period of SAEs after the end of study treatment (whether other therapies have been initiated).

1. Hospitalization

In this clinical study, the AEs that lead to hospitalization or prolonged hospitalization should be considered as SAEs. Any first hospitalization to a medical facility (even if less than 24 hours) meets this criterion.

Hospitalization does not include the following:

- Admission to rehabilitation facilities
- Admission to nursing homes
- Admission to routine emergency room
- Day surgery (such as outpatient/daytime surgery/ambulatory surgery)

Inpatient hospitalization or prolongation of existing hospitalization not associated with worsening of the AE is not an SAE per se, e.g.:

- Hospitalization for a pre-existing disease, no worsening or new AEs (e.g., hospitalization for laboratory abnormalities that occur prior to the study and still persist);
- Hospitalization due to administrative reasons (e.g., annual routine physical examination);
- On-study hospitalization as specified in the protocol (e.g., protocol-specified procedures);
- Elective hospitalization unrelated to worsening of the AEs (e.g. elective cosmetic surgery);
- Pre-scheduled treatment or surgery which should be recorded in the protocol and/or in the patient's baseline data;
- Hospitalization only for use of blood products.

Diagnostic or therapeutic invasive (e.g., surgery) and non-invasive procedures should not be reported as AEs. However, the condition that results in such procedures should be reported as an AE if it meets the AE criteria. For example, acute appendicitis that occurs during the AE reporting period should be reported as an AE, while the appendectomy thus performed should be recorded as the treatment of the AE.

1. Overdose

An overdose is defined as additionally taking the investigational product within 24 hours (adjusted according to the specific protocol), which is higher than the dose prescribed by the investigator. All overdoses during the study, whether or not related to an AE/SAE, should be reported as SAEs.

1. AEs of Special Interest

For AESIs specified in the clinical study protocol, the AESI Report Form in Hengrui Clinical Studies should be completed within 24 hours of the investigator's knowledge and reported to the sponsor.


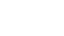
• ≥ Grade 3 infusion reaction


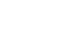
• ≥ Grade 2 diarrhoea/colitis, uveitis, and interstitial pneumonia


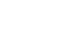
• Other ≥ Grade 3 immune-related AEs


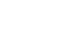
• Any possible events of abnormal liver enzymes (concurrent lack of other relevant etiology such as PD, acute viral hepatitis, cholestasis, concomitant medications, previous concurrent liver disease, etc.)

• Grade 4 amylase or lipase increased

1. Infusion Reaction

Investigators should pay close attention to possible infusion-related and/or allergic reactions, particularly acute immune-mediated adverse reactions (including cytokine storm), throughout the study.

In general, no premedication is required prior to infusion of camrelizumab. Based on the published data, allergic reactions/events are most likely to occur within 24 hours of infusion. In the event of infusion-related reactions, the infusion should be slowed or interrupted as appropriate, and supportive treatment should be given; prophylactic medication should be given prior to subsequent dosing. Possible allergic reactions may manifest as fever, chills, headache, rash, pruritus, arthralgia, hypotension or hypertension, or bronchospasm. All Grade 3 or 4 infusion reactions should be reported according to the SAE Reporting Procedure.

1. Immune-related Adverse Events (irAEs)

Immune-related adverse events (irAEs) are clinically significant side effects consistent with the immune mechanism of the investigational product. Further serological, immunological, and pathological (biopsy) data are needed to support its diagnosis. In addition, tumors, infections, metabolism, toxins, or other causes need to be excluded.

**Principles for Management of Immune-related Adverse Events (see Annex IV for details)**

**□ Immune-related Pneumonia**

Clinical studies with camrelizumab will strengthen the monitoring of signs and symptoms of immune-related pneumonia, such as cough, chest discomfort, etc. Examination will be done by imaging methods (e.g., x-rays), and high-dose hormonal therapy will be given to patients with Grade 2 or higher immune-related pneumonia. Patients with Grade 2 immune-related pneumonia may withhold camrelizumab and be treated, and patients with Grade 3 or 4 immune-related pneumonia will permanently discontinue camrelizumab.

**□ Immune-related Enteritis**

Clinical studies with camrelizumab will strengthen the monitoring of signs and symptoms of immune-related enteritis, such as abdominal pain, diarrhoea, haematochezia, etc. High-dose hormonal therapy will be given to patients with Grade 2 or higher immune-related enteritis. Patients with Grade 2 or 3 immune-related enteritis may withhold camrelizumab and be treated, and patients with Grade 4 immune-related enteritis will permanently discontinue camrelizumab.

**□ Immune-related Hepatitis**

Clinical studies with camrelizumab will strengthen the monitoring of signs and symptoms of immune-related hepatitis, such as liver discomfort, abnormal elevations of transaminases, etc. High-dose hormonal therapy will be given to patients with Grade 2 or higher immune-related enteritis. Patients with Grade 2 immune-related hepatitis may withhold camrelizumab and be treated, and patients with Grade 3 or 4 immune-related hepatitis will permanently discontinue camrelizumab.

**□ Immune-related Thyroid Dysfunction**

Abnormalities in thyroid function may occur at any time during the study, so patients will be periodically examined for thyroid function and the clinical symptoms of abnormal thyroid function will be observed in the camrelizumab study. The patient will be treated with high dose cortisone/prednisone after developing immune-related hyperthyroidism. Hormone replacement therapy will be used in the event of hypothyroidism, but glucocorticoids are not applicable.

Clinical studies with camrelizumab will strengthen the monitoring of signs and symptoms of immune-related thyroid dysfunction in patients. Patients with Grade 3 or higher immune-related thyroid dysfunction will receive high-dose hormonal therapy, and patients with Grade 4 immune-related thyroid dysfunction will permanently discontinue camrelizumab.

**□ Immune-related Nephritis and Renal Failure**

Clinical studies with camrelizumab will strengthen the monitoring of signs and symptoms of immune-related nephritis in patients. Patients with Grade 2 or higher immune-related nephritis will receive high-dose hormonal therapy, patients with Grade 2 immune-related nephritis may withhold camrelizumab and be treated, and patients with Grade 3 or 4 immune-related nephritis will permanently discontinue camrelizumab.

**□ Immune-related Hypophysitis**

Clinical studies with camrelizumab will strengthen the monitoring of signs and symptoms of immune-related hypophysitis in patients. Patients with Grade 2 or higher immune-related hypophysitis will receive high-dose hormonal therapy, patients with Grade 2 and Grade 3 immune-related hypophysitis may withhold camrelizumab and be treated, and patients with Grade 4 immune-related hypophysitis will permanently discontinue camrelizumab.

**□ Other Immune-related Adverse Reactions**

In principle, based on the severity of adverse reactions, interruption of camrelizumab is mainly adopted. When the severity recovers to Grade 1 or lower, re-use of camrelizumab may be considered. When serious Grade 3 or life-threatening Grade 4 adverse reactions occur, it should be permanently discontinued.

The management of immune-related adverse reactions should be conducted in accordance with the medical practice and guidelines of the study site. The following Table 7-2 are treatment recommendations for immune-related adverse reactions, for your information.

**Table 7-2 Treatment Recommendations for Immune-Related Adverse Reactions of Camrelizumab for Injection**

| **CTCAE Grade** | **Clinical Management *** | **Camrelizumab Treatment** |
| --- | --- | --- |
| Grade 1 (mild) | - Close observation, especially diarrhoea  • Supportive care measures | No change |
| Grade 2 (moderate) | - Close monitoring  • Supportive care measures  • Topical steroids, i.e., for skin reactions/colitis, if the symptoms persist ≥ 7 days, start to give 1 mg/kg prednisone or equivalents; if the symptoms worsen, give 1 mg/kg prednisone IV or orally | Interrupt the dosing. After the reaction recovers to ≤ Grade 1, re-administer |
| ≥ Grade 3 (severe) * | -2 mg/kg prednisone | Permanently discontinue |

* In case of Grade 3 topical skin and endocrine disease, the treatment may not be discontinued, because it can often heal (skin) or be treated with alternative therapy (endocrine) (Weber, Jeffrey S. MD, PhD, et al., “Toxicities of immunotherapy for the practitioner,” J Clin Oncol, April 2015)

1. SAE Reporting Procedure

SAEs should be reported from the time the patient signs the ICF until 30 calendar days (inclusive) after the last dose of investigational product. During the study, any SAE must be reported to the clinical monitor and principal investigators within 24 hours who should complete, sign and date the Serious Adverse Event (SAE) Report Form for Clinical Study of New Drugs, which should be immediately reported via fax to the sponsor, the leading site, the Ethics Committee of the study site, China Food and Drug Administration (CFDA) and the drug administration in the investigator’s region (province or city).

Any SAE occurs during continued dosing after the end of study also must be reported to the sponsor within 24 hours. Information about all SAEs should be recorded on the Serious Adverse Event Report Form. SAEs that occur during continued dosing up to 30 days after the last dose must be reported. SAEs that occur after 30 days of the last dose are generally not reported unless suspected to be related to the investigational product.

SAEs should be recorded in detail, including the symptoms, severity, onset date, duration of treatment, action taken, time and manner of follow-up, and outcomes. If, in the opinion of the investigator, an SAE is not related to the investigational product but potentially related to the study conditions (e.g., termination of the pre-existing treatment, or comorbidities during the trial), details should be elaborated in the narrative section of the SAE page of the CRF. If the severity of an ongoing SAE or its relationship to the investigational product is changed, the SAE follow-up report should be sent to the sponsor immediately. All SAEs should be followed until recovery or stabilization.

## 7.6 Management Comments for Adverse Events

**7.6.1 Immune-related Adverse Events (irAEs)**

Immune-related adverse events (irAEs) are clinically significant side effects consistent with the immune mechanism of the investigational product. Further serological, immunological, and pathological (biopsy) data are needed to support its diagnosis. In addition, tumors, infections, metabolism, toxins, or other causes need to be excluded.

**Principles for Management of Immune-related Adverse Events (see Annex I for details):**

The management of immune-related adverse reactions should be conducted in accordance with the medical practice and guidelines of the study site. The following are treatment recommendations for immune-related adverse reactions, see Annex I for details.

Patients treated with corticosteroids should receive calcium and vitamin D3, acid suppression and protection of gastric mucosa.

- **Immune-related Pneumonia**

Clinical studies with camrelizumab will strengthen the monitoring of signs and symptoms of immune-related pneumonia, such as cough, chest discomfort, etc. Examination will be done by chest CT, and high-dose hormonal therapy will be given to patients with Grade 2 or higher immune-related pneumonia. Patients with Grade 2 immune-related pneumonia may withhold camrelizumab and be treated, and patients with Grade 3 or 4 immune-related pneumonia will permanently discontinue camrelizumab. Respiratory consultation is recommended.

Refer to the following recommendations for specific operations:

Grade 2 pneumonia: give 1 mg/kg/day methylprednisolone IV or orally at the equivalent dose, and closely monitor CT changes. After recovery to Grade 1, continue to give 0.5 mg/kg/day prednisone orally for 2 weeks, and then reduce 5 mg prednisone weekly, until discontinuation.

Grade 3 pneumonia: give 2-4 mg/kg/day methylprednisolone IV or at the equivalent dose by intravenous injection, and closely monitor CT changes. After recovery to Grade 1, reduce the dose by 50% every 3 days, give 0.5 mg/kg/day prednisone orally for 2 weeks, and then reduce 5 mg prednisone weekly, until discontinuation.

If the symptoms do not improve or worsen for 3-5 days by hormone therapy, immunosuppressant therapy may be combined after communication with the sponsor.

- **Immune-related Hepatitis**

Clinical studies with camrelizumab will strengthen the monitoring of signs and symptoms of immune-related hepatitis, such as liver discomfort, abnormal elevations of transaminases, etc. High-dose hormonal therapy will be given to patients with Grade 2 or higher immune-related hepatitis. Refer to the following recommendations for specific operations:

Grade 2 hepatitis: give 0.5-1 mg/kg/day methylprednisolone IV or at the equivalent dose orally, and closely monitor changes in liver function parameters. After recovery to Grade 1, slowly reduce the hormone over not less than 1 month.

Grade 3 hepatitis: give 1-2 mg/kg/day methylprednisolone IV or at the equivalent dose by intravenous injection, and closely monitor changes in liver function parameters. After recovery to Grade 1, slowly reduce the hormone over not less than 1 month. If the symptoms do not improve or worsen for 3-5 days by hormone therapy, immunosuppressant therapy may be combined after communication with the sponsor.

- **Immune-related Enteritis**

Clinical studies with camrelizumab will strengthen the monitoring of signs and symptoms of immune-related enteritis, such as abdominal pain, diarrhoea, haematochezia, etc. High-dose hormonal therapy will be given to patients with Grade 2 or higher immune-related hepatitis. Patients with Grade 2 or 3 immune-related enteritis may withhold camrelizumab and be treated, and patients with Grade 4 immune-related enteritis will permanently discontinue camrelizumab.

- **Immune-related Thyroid Dysfunction**

Abnormalities in thyroid function may occur at any time during the study, so patients will be periodically examined for thyroid function and the clinical symptoms of abnormal thyroid function will be observed in the camrelizumab study. The patient will be treated with high dose cortisone/prednisone after developing immune-related hyperthyroidism. Hormone replacement therapy will be used in the event of hypothyroidism, but glucocorticoids are not applicable.

Clinical studies with camrelizumab will strengthen the monitoring of signs and symptoms of immune-related thyroid dysfunction in patients. Patients with Grade 3 or higher immune-related thyroid dysfunction will receive high-dose hormonal therapy, and patients with Grade 4 immune-related thyroid dysfunction will permanently discontinue camrelizumab.

- **Immune-related Nephritis and Renal Failure**

Clinical studies with camrelizumab will strengthen the monitoring of signs and symptoms of immune-related nephritis in patients. Patients with Grade 2 or higher immune-related nephritis will receive high-dose hormonal therapy, patients with Grade 2 immune-related nephritis may withhold camrelizumab and be treated, and patients with Grade 3 or 4 immune-related nephritis will permanently discontinue camrelizumab.

- **Immune-related Hypophysitis**

Clinical studies with camrelizumab will strengthen the monitoring of signs and symptoms of immune-related hypophysitis in patients. Patients with Grade 2 or higher immune-related hypophysitis will receive high-dose hormonal therapy, patients with Grade 2 and Grade 3 immune-related hypophysitis may withhold camrelizumab and be treated, and patients with Grade 4 immune-related hypophysitis will permanently discontinue camrelizumab.

- **Other Immune-related Adverse Reactions**

In principle, based on the severity of adverse reactions, interruption of camrelizumab is mainly adopted. When the severity recovers to Grade 1 or lower, re-use of camrelizumab may be considered. When serious Grade 3 or life-threatening Grade 4 adverse reactions occur, it should be permanently discontinued.

**7.6.2 Infusion Reactions**

As camrelizumab is a fully humanized monoclonal antibody, it is unlikely to cause infusion-related reactions, and thus requires no premedication before infusion. In the event of infusion-related reactions, the infusion should be slowed down or interrupted as appropriate, and supportive treatment should be given, and premedication should be given prior to subsequent dosing. Related symptoms and signs of acute infusion-related reactions (including cytokine release syndrome, angioedema, anaphylactic shock and allergic reactions; refer to Common Terminology Criteria for Adverse Events, NCI CTCAE v5.0) usually occur during or immediately after drug infusion, and disappear within 24 hours after the completion of infusion. Symptoms and signs include allergic reactions/hypersensitivity (including drug-induced fever), cough, fear of cold, chills/shivering, dizziness, headache, tiredness (fatigue, somnolence), rash/peeling, skin itching/pruritus, joint pain, muscle pain, low or high blood pressure, nausea, vomiting, sweating, tachycardia, tumor pain, urticaria (rubella), dyspnea (shortness of breath) or bronchial spasm. All Grade 3 or 4 infusion-related reactions should be reported to the sponsor within 24 h and reported as SAEs if they meet the criteria for SAEs.

The management of allergic reactions should be based on the medical practice and guidelines of the study site. The following are recommendations for the treatment of infusion-related reactions for reference.

Table 7-3 Treatment Recommendations for Infusion Reactions

| **CTCAE Grade** | **Clinical Symptoms** | **Clinical Management** | **Camrelizumab Treatment** |
| --- | --- | --- | --- |
| Grade 1 (mild) | Mild transient reactions; | Bedside observation, close monitoring until recovery. (Prophylactic medication is recommended prior to subsequent infusions: diphenhydramine 50 mg, or equivalent and/or acetaminophen 325-1000 mg, at least 30 min before camrelizumab administration. | No change |
| Grade 2 (moderate) | Moderate reactions, treatment or infusion interruption indicated but respond promptly to symptomatic treatment (e.g., antihistamines, NSAIDS, narcotics, bronchodilators, intravenous infusion etc. | Intravenous infusion of normal saline, diphenhydramine 50 mg IV or equivalent and/or acetaminophen 325-1000 mg; bedside observation, close monitoring until recovery. According to clinical needs, consider corticosteroids or bronchodilators;  The amount of infused investigational product will be recorded in the original medical record; prophylactic medication is recommended prior to subsequent infusions: at least 30 min before camrelizumab administration, diphenhydramine 50 mg or equivalent and/or acetaminophen 325-1000 mg. If necessary, corticosteroids (at a dose equivalent to 25 mg of hydrocortisone). | Interrupt the dosing. Restart the medication at 50% of the initial infusion rate after the symptoms disappear. If there is no complication within 30 min, the infusion rate can be increased to 100% of the original infusion rate. Closely monitor the patient. If symptoms recur, no longer infuse camrelizumab for the current treatment. |
| ≥ Grade3 (severe) | Grade 3: severe reactions, which do not immediately respond to therapy and/or dose interruption; or the symptoms reoccur after resolution; sequelae requiring hospitalization. Grade 4: life-threatening | • Immediately discontinue the infusion of camrelizumab; start the IV infusion of normal saline.  • Bronchodilators: 0.2-1 mg adrenaline in 1:1000 solution via subcutaneous injection, or slow infusion injection of 0.1-0.25 mg adrenaline in 1:10000 solution and/or, if necessary, diphenhydramine 50 mg + methylprednisolone 100 mg or equivalent via intravenous injection;  • Follow research institute guidelines for the treatment of allergic reactions; bedside observation, close monitoring until recovery. | Permanent discontinue. |

Note: consult with a specialist if necessary to assist in diagnosis and treatment.

# 8. Statistical Methods

## 8.1 Statistical Analysis Datasets

Full Analysis Set (FAS): includes all subjects who signs the ICF and take at least one dose of investigational product, according to the intention-to-treat (ITT) principle. FAS is the primary analysis set for efficacy analysis in this study.

Per-protocol Set (PPS): a more protocol-compliant subset of FAS; subjects with major protocol deviations judged to have a significant impact on the primary efficacy analysis will be excluded from the PPS. Missing data will not be imputed. The study efficacy endpoints will be statistically analyzed using both the FAS and PPS.

Safety Analysis Set (SAS): includes all enrolled subjects who take at least one dose of investigational product and have safety variables recorded. SAS will be used for safety analysis.

## 8.2 Statistical Analysis Plan

The results of this study will be mainly analyzed by descriptive statistical methods. The mean, standard deviation, median, maximum and minimum are presented for measurement data, and the frequency (constituent ratio), rate and its confidence interval are presented for enumeration data and grade data.

All statistical analyses will be programmed and calculated using SAS 9.2. All statistical tests will be performed using a two-sided test, with P < 0.05 considered as statistically significant, and 95% CI will be applied.

**8.2.1 Efficacy Analysis**

The ORR and DCR along with their 95% confidence intervals (CIs) will be calculated using the Clopper-Pearson method. PFS, EFS, and OS curves will be plotted by the Kaplan-Meier method, and their 95% CIs will be calculated by the Brookmeyer-Crowley method. Subgroup analysis of ORR will be performed based on baseline patient characteristics. The survival curves of the different subgroups of patients will be compared using the log-rank test. Categorical variables will be compared using Fisher’s exact test. Comparisons of unpaired continuous variables will be performed using the unpaired Wilcoxon test, and Wilcoxon paired test will be used for paired samples. The statistical analyses will be performed using SAS 9.4, and P < 0.05 will be considered statistically significant.

**8.2.2 Safety Evaluation**

Treatment-emergent adverse events (TEAEs) and treatment-related adverse events (TRAEs) will be listed mainly by descriptive statistical analysis, including assessment of relationship as "definitely related", "possibly related" and "not judged". For the results of laboratory tests, the conditions that are normal before the study but abnormal after treatment and the causality with the investigational product when abnormal changes occur will be described. Chi-square test will be performed on the incidence of related adverse reactions between different stratification groups.

# 9. Quality Control and Quality Assurance

The study personnel must be physicians trained in clinical studies and work under the guidance of senior professionals.

Before a study, the clinical ward must meet the requirements of standardization and ensure that rescue equipment is complete.

It is recommended that the patients be given drugs by professional nursing staff to understand the medication in detail, and to ensure the compliance of the patients.

The investigator must truthfully complete the case report form.

The investigator should follow standard operating procedures to supervise the conduct of the clinical study, confirm that all data records and reports are correct and complete and all CRFs are completed correctly and consistent with the original data, and ensure that the study is conducted in accordance with the clinical study protocol;

In the event of an SAE, the EC should be notified in a timely manner, and the study should be temporarily discontinued if necessary.

# 10. Ethical, Regulatory and Administrative Principles

## 10.1 Ethical Principles

This study will be conducted in accordance with the principles established by the 18^th^ World Medical Assembly (Helsinki, 1964) and all subsequent amendments.

## 10.2 Laws and Regulations

This study will be conducted in accordance with all laws and regulations.

## 10.3 Data Protection

The personal data of patients and investigators that may be included in the EDC system should be treated in accordance with all applicable local laws and regulations.

When archiving or processing the personal data related to the investigator and/or patient, the study director or principal investigator should take all appropriate measures to protect and prevent access to such data by any unauthorized third party.

## 10.4 Confidentiality Agreement

All materials, information (oral or written) and unpublished documents, including this protocol and CRF, provided to the investigators (or any action taken by the sponsor on behalf of the investigator), may not be disclosed to unauthorized personnel by the investigators or sub-site's study personnel without the prior formal written consent of the leading site.

The investigator should keep confidentiality of all information received, obtained, or derived during the course of this study, except those permitted by regulations, and should take all necessary procedures to ensure that confidentiality is not disclosed.

## 10.5 Record Retention

The investigator should be responsible for the retention of study documents until end of the study. In addition, the investigator should comply with the specific local regulations/guidelines regarding patient record retention.

Unless otherwise specified in the Investigator Agreement, the investigators are advised to keep the study documents for at least five years after completion or discontinuation of the study in accordance with other standards and/or local laws.

## 10.6 Early Study Discontinuation

The sponsor may decide to discontinue the study at any time and for any reason; the decision to discontinue the study will be communicated in writing to the sub-site investigators.

Similarly, if the sub-site investigator decides to withdraw from the study, the sponsor must be notified in writing.

The EC/IRB and health authorities should be notified, as applicable, in accordance with local regulations.

## 10.7 Sponsor Audits and Inspections by Regulatory Authorities

The investigators at each site agrees to allow direct access to the patient’s study records by sponsor auditors/inspectors from regulatory authorities for review, and understands that these personnel are subject to the principle of professional confidentiality and therefore will not disclose any personal identity or medical information of the patient.

The investigator at each site will make every effort to assist in the audits and inspections so that auditors/inspectors have access to all necessary devices, data, and documents.

During these inspections, the confidentiality of verification data and the protection of patients should be respected.

The investigator at each site should communicate the results and information given by the regulatory authorities after the inspection to the sponsor immediately.

At the request of the sponsor, the investigator at each site should take appropriate measures to take corrective actions for all problems found during the audit or inspection.

# 11. Protocol Amendments

Any amendments to the protocol will be documented in a written amendment signed by the investigator at each site. The signed amendment will be attached to this protocol.

Amendments to this protocol may be submitted in accordance with local regulations.

# 12. Use of Documentation and Study Results

## 12.1 Ownership and Use of Study Data and Study Results

The sponsor has full access to the final data to conduct appropriate academic analysis and reporting of the study results.

## 12.2 Publication

Any subsequent presentation or publication by study participants (including sub-studies) must be approved by the sponsor and refer to this study and its first publication.

The sponsor may request that the name of the sponsor and/or the name(s) of one or more employees of the sponsor be listed or not listed in this publication.

The sponsor may delay the publication or presentation for a limited period of time in order to protect the confidentiality or ownership of any information contained therein.

# 13. Clinical Study Progress

The study is expected to last 30 months, including an 18-month enrollment period.

- First patient enrolled/start of study: 01 June 2020
- Last patient enrolled: 01 December 2021
- End of study: December 2022 (more than 1 year after last eligible patient enrolled)
- Database lock date: February 2023
- Report date: February 2023

# Note: The change of the actual duration of study is not a protocol violation.

# References

1. Globecan 2018；
2. CSCO Guidelines for Diagnosis and Treatment of Gastric Cancer, 2019;
3. Nature. 2014; 513(7517): 202-209.
4. Chen DS, Mellman I. Immunity. 2013. 25;39(1):1-10.
5. Wolchock et al, J Clin Oncol 2013 ASCO Annual Meeting Abstracts 31:15_suppl.
6. Tabernero J, et al. Oral presentation at ASCO 2019
7. Kang YK et al. Poster presentation at ESMO 2017.
8. Sha Zhao et al., Cancer Immunol Res. 2019; pii: canimm.0640.2017
9. Lin Shen, et al. J Clin Oncol 37, 2019 (suppl; abstr 4031)

# Annex I Principles for Management of Immune-related Adverse Events

1. Principles for Management of Gastrointestinal Adverse Events


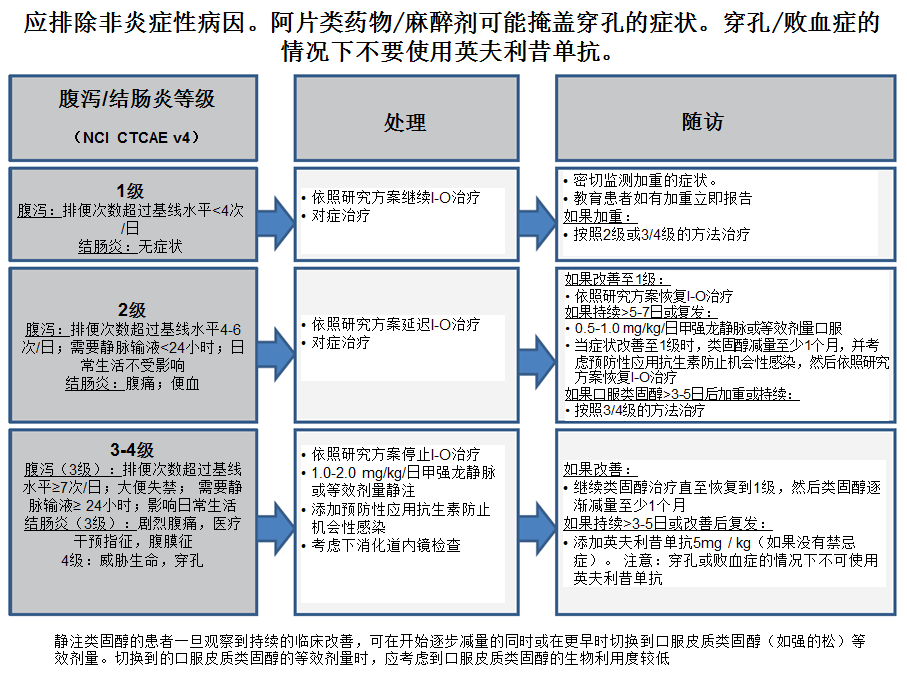


**Rule out non-inflammatory causes.** **Opioids/anesthetics may mask the symptoms of perforation.** **Infliximab should not be used in case of perforation or sepsis.**

Once sustained clinical improvement is observed with IV steroids, patients may start to reduce the dose and switch to an equivalent dose of oral corticosteroids (e.g., prednisone) simultaneously or earlier. Low bioavailability of oral corticosteroids should be taken into account when switching to an equivalent dose of oral corticosteroids

**Diarrhoea/colitis grade**

**Management**

**Follow-up**

- Continue I-O treatment per protocol
- Symptomatic treatment
- Delay I-O treatment per protocol
- Symptomatic treatment
- Discontinue I-O treatment per protocol
- 1.0-2.0 mg/kg/day methylprednisolone IV or at the equivalent dose by intravenous injection
- Add prophylactic antibiotics to prevent opportunistic infections
- Consider lower gastrointestinal endoscopy

**Grade 1**

Diarrhoea: bowel movements < 4 times/day above baseline

Colitis: asymptomatic

**Grade 2**

Diarrhoea: bowel movements 4-6 times/day above baseline; IV infusion < 24 hours required; daily living unaffected

Colitis: abdominal pain; hematochezia

**Grade 3-4**

Diarrhoea (Grade 3): bowel movements ≥ 7 times/day above baseline; fecal incontinence; IV infusion ≥ 24 hours required; affecting daily living

Colitis (Grade 3): severe abdominal pain, medical intervention indicated, peritoneal sign

Grade 4: life-threatening, perforation

Closely monitor for worsening symptoms.

- Educate patients to report any exacerbation immediately

If the symptoms worsen:

- Treat as Grade 2 or 3/4

If the symptoms improve to Grade 1:

- Resume I-O treatment per protocol

If the symptoms persist > 5-7 days or relapse:

- 0.5-1.0 mg/kg/day methylprednisolone IV or at the equivalent dose orally
- If the symptoms improve to Grade 1, taper steroids over at least 1 month, consider prophylactic antibiotics to prevent opportunistic infections, and then resume I-O treatment per protocol

If the symptoms worsen or persist after > 3-5 days of oral steroids:

- Treat as Grade 3/4

If the symptoms improve:

- Continue steroid therapy until recovery to Grade 1, and then taper steroids over at least 1 month

If the symptoms persist > 3-5 days or relapse after improvement:

- Add infliximab 5 mg/kg (if not contraindicated). Caution: Infliximab should not be used in case of perforation or sepsis.

2. Principles for Management of Pulmonary Adverse Events


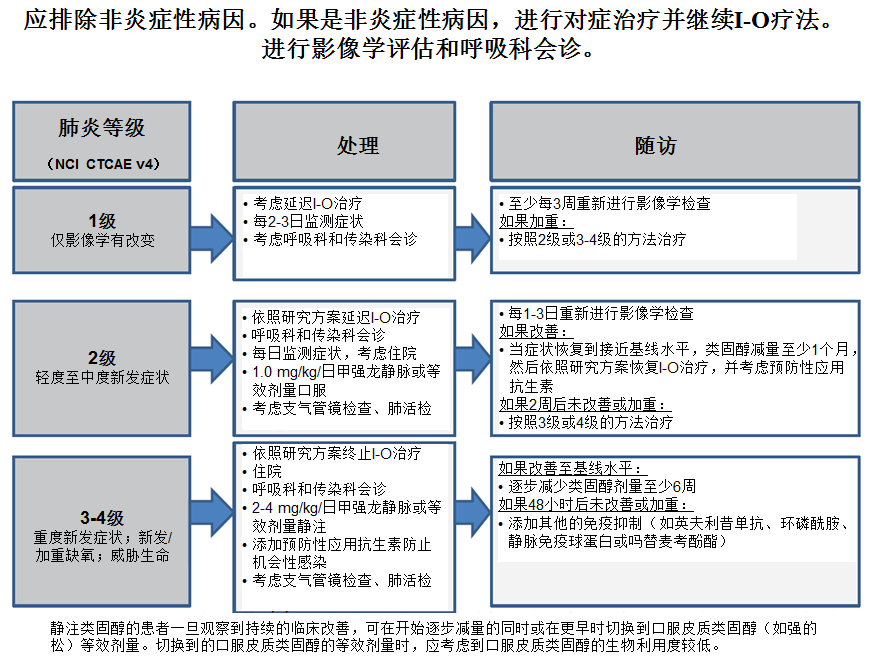


**Rule out non-inflammatory causes.** **If non-inflammatory cause, give symptomatic treatment and continue I-O treatment.**

**Conduct radiographic assessment and consultation with respiratory department.**

**Pneumonia Grade**

**Management**

**Follow-up**

**Grade 1**

Radiographic changes only

**Grade 2**

Mild to moderate new symptoms

**Grade 3-4**

Severe new symptoms; new/worsening hypoxia; life-threatening

Once sustained clinical improvement is observed with IV steroids, patients may start to reduce the dose and switch to an equivalent dose of oral corticosteroids (e.g., prednisone) simultaneously or earlier. Low bioavailability of oral corticosteroids should be taken into account when switching to an equivalent dose of oral corticosteroids.

- Consider delay of I-O treatment
- Monitor symptoms every 2-3 days
- Consider consultation with respiratory department and department of infectious diseases
- Delay I-O treatment per protocol
- Consultation with respiratory department and department of infectious diseases
- Monitor symptoms daily, consider hospitalization
- 1.0 mg/kg/day methylprednisolone IV or at the equivalent dose orally
- Consider bronchoscopy and lung biopsy
- Discontinue I-O treatment per protocol
- Hospitalization
- Consultation with respiratory department and department of infectious diseases
- 2-4 mg/kg/day methylprednisolone IV or at the equivalent dose by intravenous injection
- Add prophylactic antibiotics to prevent opportunistic infections
- Consider bronchoscopy and lung biopsy

• Repeat imaging at least every 3 weeks

If the symptoms worsen:

· Treat as Grade 2 or Grade 3-4

- Repeat imaging every 1-3 days

If the symptoms improve:

- If the symptoms return to near baseline, reduce steroids over at least 1 month, then resume I-O treatment per protocol and consider prophylactic antibiotics

If the symptoms do not improve or worsen after 2 weeks:

- Treat as Grade 3 or 4

If the symptoms improve to baseline:

- Taper steroids over at least 6 weeks

If the symptoms do not improve or worsen after 48 hours:

- Add additional immunosuppressants (eg, infliximab, cyclophosphamide, intravenous immunoglobulin, or mycophenolate mofetil)

1. Principles for Management Hepatic Adverse Events


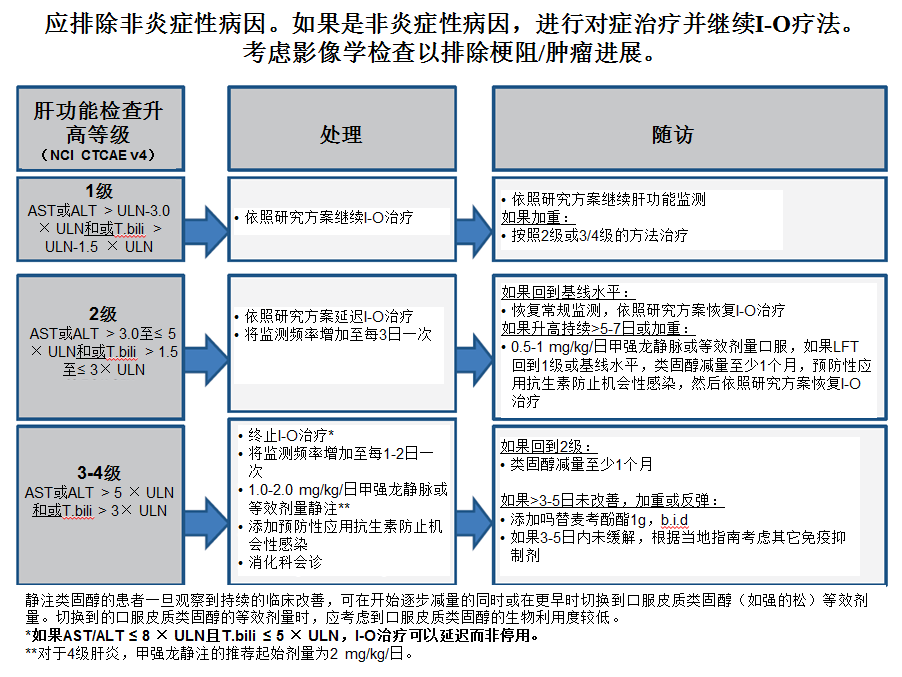


Once sustained clinical improvement is observed with IV steroids, patients may start to reduce the dose and switch to an equivalent dose of oral corticosteroids (e.g., prednisone) simultaneously or earlier. Low bioavailability of oral corticosteroids should be taken into account when switching to an equivalent dose of oral corticosteroids.

*** If AST/ALT ≤ 8 × ULN and total bilirubin ≤ 5 × ULN, I-O treatment may be delayed rather than discontinued.**

** For Grade 4 hepatitis, the recommended starting dose of IV methylprednisolone is 2 mg/kg/day.

**Grade of liver function test results increased**

**Management**

**Follow-up**

**Grade 1**

AST or ALT > ULN-3.0 x ULN and/or total bilirubin > ULN-1.5 x ULN

**Grade 2**

AST or ALT > 3.0 to ≤ 5 × ULN and/or total bilirubin > 1.5 to ≤ 3 × ULN

**Grade 3-4**

AST or ALT > 5 x ULN and/or total bilirubin > 3 x ULN

- Continue I-O treatment per protocol
- Delay I-O treatment per protocol
- Increase the frequency of monitoring to every 3 days
- Discontinue I-O treatment*
- Increase the frequency of monitoring to every 1-2 days
- 1.0-2.0 mg/kg/day methylprednisolone IV or at the equivalent dose by intravenous injection**
- Add prophylactic antibiotics to prevent opportunistic infections
- Consult with gastrointestinal department

• Continue monitoring the liver function per protocol

If the symptoms worsen:

· Treat as Grade 2 or 3/4

If recovery to baseline:

- Resume routine monitoring and resume I-O treatment per protocol

If the increases persist for > 5-7 days or worsen:

- 0.5-1 mg/kg/day methylprednisolone IV or at the equivalent dose orally. If LFT returns to Grade 1 or baseline, taper steroids over at least 1 month, give prophylactic antibiotics to prevent opportunistic infections, and then resume I-O treatment per protocol

If recovery to Grade 2:

- Taper steroids over at least 1 month

If the increases do not improve, worsen or rebound after > 3-5 days:

- Add mycophenolate mofetil 1 g, b.i.d
- If not resolved within 3-5 days, consider other immunosuppressants according to local guidelines

**Rule out non-inflammatory causes.** **If non-inflammatory cause, give symptomatic treatment and continue I-O treatment.**

**Consider imaging to rule out obstruction/tumor progression.**

1. Principles for Management of Endocrine Adverse Events


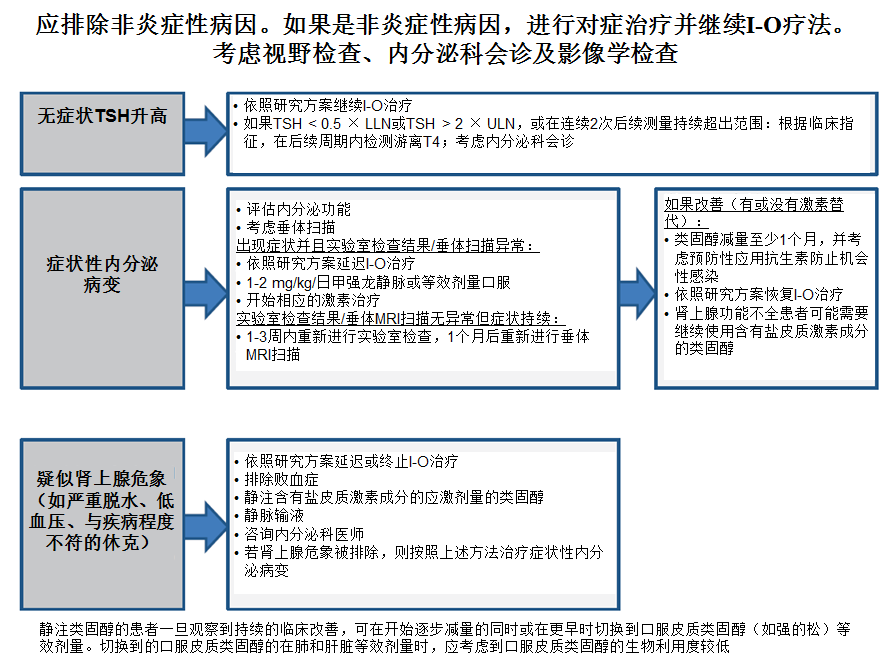


**Rule out non-inflammatory causes.** **If non-inflammatory cause, give symptomatic treatment and continue I-O treatment.**

**Consider visual field test, consultation with the endocrinology department, and imaging.**

**Asymptomatic TSH increased**

**Symptomatic endocrine disorder**

**Suspected adrenal crisis (e.g., severe dehydration, hypotension, shock inconsistent with disease severity)**

Once sustained clinical improvement is observed with IV steroids, patients may start to reduce the dose and switch to an equivalent dose of oral corticosteroids (e.g., prednisone) simultaneously or earlier. Low bioavailability of oral corticosteroids should be taken into account when switching to an equivalent dose of oral corticosteroids in the lungs and liver.

- Continue I-O treatment per protocol
- If TSH < 0.5 × LLN or TSH > 2 × ULN, or if 2 subsequent consecutive measurements continue to be out of range: test the free T4 in subsequent cycles as clinically indicated; consider consultation with the endocrinology department
- Delay or discontinue I-O treatment per protocol
- Rule out sepsis
- Intravenous injection of stress dose of steroids containing mineralocorticoid component
- Intravenous infusion
- Consult with an endocrinologist
- If adrenal crisis is ruled out, treat symptomatic endocrine lesions as described above
- Evaluate endocrine function
- Consider pituitary scan

Symptomatic and abnormal laboratory/pituitary scan:

- Delay I-O treatment per protocol
- 1-2 mg/kg/day methylprednisolone IV or at the equivalent dose orally
- Initiate appropriate hormonal therapy

Normal laboratory results/pituitary MRI scan but symptoms persist:

- Repeat the laboratory tests within 1-3 weeks and pituitary MRI after 1 month

If improved (with or without hormone replacement):

- Taper steroids over at least 1 month and consider prophylactic antibiotics to prevent opportunistic infections
- Resume I-O treatment per protocol
- Patients with adrenal insufficiency may need to continue steroids with mineralocorticoid component

5. Principles for Management of Skin Adverse Events


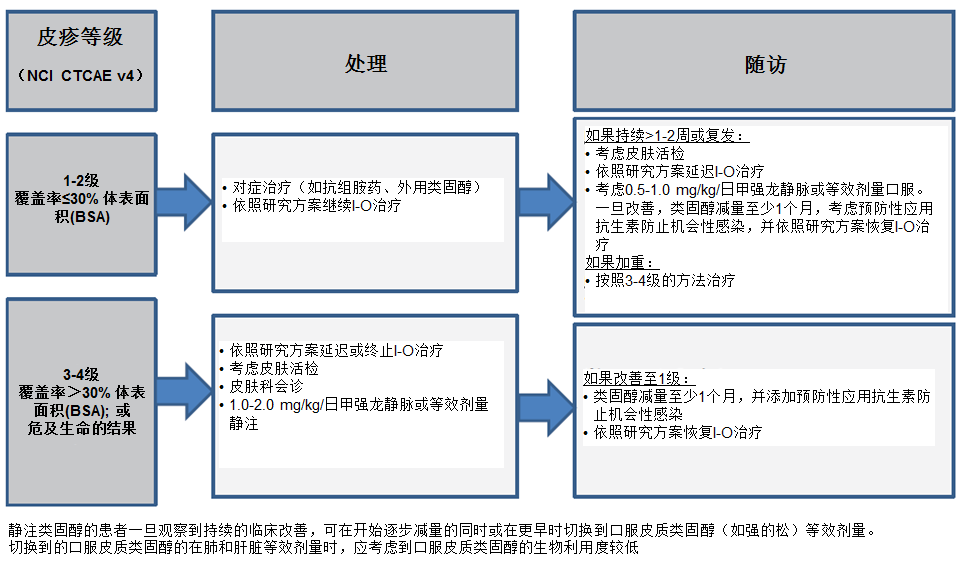


**Management**

**Follow-up**

**Rash grade**

**Grade 1-2**

**Coverage ≤ 30% of body surface area (BSA)**

**Grade 3-4**

**Coverage > 30% of body surface area (BSA); or life-threatening outcome**

Once sustained clinical improvement is observed with IV steroids, patients may start to reduce the dose and switch to an equivalent dose of oral corticosteroids (e.g., prednisone) simultaneously or earlier. Low bioavailability of oral corticosteroids should be taken into account when switching to an equivalent dose of oral corticosteroids in the lungs and liver.

- Symptomatic treatment (e.g., antihistamines, topical steroids)
- Continue I-O treatment per protocol
- Delay or discontinue I-O treatment per protocol
- Consider skin biopsy
- Consultation with the dermatology department
- 1.0-2.0 mg/kg/day methylprednisolone IV or at the equivalent dose by intravenous injection

If persist > 1-2 weeks or recur:

- Consider skin biopsy
- Delay I-O treatment per protocol
- Consider 0.5-1.0 mg/kg/day methylprednisolone IV or at the equivalent dose orally. Once improved, taper steroids over at least 1 month, consider prophylactic antibiotics to prevent opportunistic infections, and resume I-O treatment per protocol

If the symptoms worsen:

- Treat as Grade 3-4

If the symptoms improve to Grade 1:

- Taper steroids over at least 1 month and add prophylactic antibiotics to prevent opportunistic infections
- Resume I-O treatment per protocol

*（ Weber JS, Postow M, Lao CD, Schadendorf D. Management of Adverse Events Following Treatment With Anti- Programmed Death-1 Agents. Oncologist. 2016 Jul 8: 2016-005*
